# Genome-scale metabolic modeling of the human milk oligosaccharide utilization by *Bifidobacterium longum* subsp. *infantis*

**DOI:** 10.1128/msystems.00715-23

**Published:** 2024-02-16

**Authors:** Loreto Román, Felipe Melis-Arcos, Tomás Pröschle, Pedro A. Saa, Daniel Garrido

**Affiliations:** 1 Department of Chemical and Bioprocess Engineering, School of Engineering, Pontificia Universidad Católica de Chile, Santiago, Chile; 2 Institute for Mathematical and Computational Engineering, Pontificia Universidad Católica de Chile, Vicuña Mackenna, Santiago, Chile; University of California San Diego, La Jolla, California, USA

**Keywords:** genome-scale metabolic models, GSMM, *Bifidobacterium longum *subsp.*infantis*, infant gut microbiome, bacterial metabolism

## Abstract

*Bifidobacterium longum* subsp. *infantis* is a representative and dominant species in the infant gut and is considered a beneficial microbe. This organism displays multiple adaptations to thrive in the infant gut, regarded as a model for human milk oligosaccharides (HMOs) utilization. These carbohydrates are abundant in breast milk and include different molecules based on lactose. They contain fucose, sialic acid, and N-acetylglucosamine. *Bifidobacterium* metabolism is complex, and a systems view of relevant metabolic pathways and exchange metabolites during HMO consumption is missing. To address this limitation, a refined genome-scale network reconstruction of this bacterium is presented using a previous reconstruction of *B. infantis* ATCC 15967 as a template. The latter was expanded based on an extensive revision of genome annotations, current literature, and transcriptomic data integration. The metabolic reconstruction (*i*LR578) accounted for 578 genes, 1,047 reactions, and 924 metabolites. Starting from this reconstruction, we built context-specific genome-scale metabolic models using RNA-seq data from cultures growing in lactose and three HMOs. The models revealed notable differences in HMO metabolism depending on the functional characteristics of the substrates. Particularly, fucosyl-lactose showed a divergent metabolism due to a fucose moiety. High yields of lactate and acetate were predicted under growth rate maximization in all conditions, whereas formate, ethanol, and 1,2-propanediol were substantially lower. Similar results were also obtained under near-optimal growth on each substrate when varying the empirically observed acetate-to-lactate production ratio. Model predictions displayed reasonable agreement between central carbon metabolism fluxes and expression data across all conditions. Flux coupling analysis revealed additional connections between succinate exchange and arginine and sulfate metabolism and a strong coupling between central carbon reactions and adenine metabolism. More importantly, specific networks of coupled reactions under each carbon source were derived and analyzed. Overall, the presented network reconstruction constitutes a valuable platform for probing the metabolism of this prominent infant gut bifidobacteria.

**IMPORTANCE:**

This work presents a detailed reconstruction of the metabolism of *Bifidobacterium longum* subsp. *infantis*, a prominent member of the infant gut microbiome, providing a systems view of its metabolism of human milk oligosaccharides.

## INTRODUCTION

*Bifidobacterium* species are early colonizers of the infant gut microbiota (1, 2). This genus includes Gram-positive, rod-shaped anaerobic, and nonmotile bacteria that are usually regarded as beneficial for the host (3, 4). Certain bifidobacteria are used as probiotics in the food industry for their multiple health-promoting activities (5). These microorganisms have a saccharolytic lifestyle, fermenting several carbohydrates with a preference for oligo and dietary polysaccharides (6).

The *Bifidobacterium* genus relies on the bifid shunt for central metabolism (7). This pathway is characterized by the presence of fructose-6-phosphate phosphoketolase (PKL), and it enables *Bifidobacterium* to produce more ATP per fermented glucose (2.5 ATP) than fermentative pathways present in other bacteria, e.g., two ATP in lactic acid bacteria (8). Essential enzymes in this shunt are fructose 6-phosphate erythrose 4-phosphate lyase (F6PE4PL), transaldolase (TALA), and transketolases (TK1 and TKT2), as well as ribose 5-phosphate isomerase and epimerase (6). *Bifidobacterium* metabolism theoretically produces acetate and lactate in a 3:2 ratio as end-products from hexoses (9). This ratio is indeed variable, and it depends on the nature of the substrate used for fermentation (10). The production of lactate and ethanol is useful for NADH regeneration (11), and the TCA cycle is incomplete in most *Bifidobacterium* (12). In addition, *Bifidobacterium* genomes display an enrichment in carbohydrate metabolism genes (13–15). These include exo- and endoglycolytic enzymes that target complex carbohydrates, ABC transporters, and feeder pathways deriving carbohydrates into the bifid shunt (16–18). Genomic and functional studies have shown that these microorganisms have coevolved with the host and display an extensive array of adaptations for the infant gut environment (19, 20).

Human milk contains high concentrations of human milk oligosaccharides (HMOs) compared to other mammals (21). They represent a diverse family of structures with a degree of polymerization from 3 to 15 (22). HMOs represent a high-energy investment from the mother, aimed at providing the newborn with a healthy microbiota dominated by *Bifidobacterium* species (23). Some HMOs contain sialic acid residues (NeuAc; e.g., 6'-sialyllactose; 6′SL), fucose (Fuc; e.g., 3'-fucosyllactose; 3′FL), or nondecorated with these residues (e.g., lacto-N-neotetraose; Galβ1–4GlcNAcβ1–4Galβ1–4Glc; LNnT, among others) (18). Several infant gut-associated bifidobacteria specialize in HMO consumption (24, 25). *Bifidobacterium longum* subsp. *infantis* (*B. infantis*) is one of the most studied microorganisms for its ability to utilize different HMOs (26, 27). It possesses several genomic and functional adaptations for intracellularly accessing and degrading small HMOs (3′FL and 6′SL) or longer ones (28). *B. infantis* contains multiple ABC transporters that import HMOs and various glycoside hydrolases, including α-fucosidases and α-sialidases (17, 29). These genomic patterns are well conserved in the subspecies *infantis* (30–32) and described in other species such as *Bifidobacterium breve* and some strains of *B. longum* subsp. *longum* (18).

Genome-scale metabolic models (GSMMs) are powerful mathematical structures that provide a coherent representation of cell metabolism, enabling a better understanding of metabolic behaviors and microbial adaptations under different scenarios (33, 34). To improve their prediction capabilities, computational methods integrating additional omics data, such as transcriptomics, have been developed and evaluated (35, 36). These tools, paired with high-quality metabolic models, have provided a helpful platform for probing cellular metabolism and increasing our knowledge about bifidobacterial metabolism (7, 12, 37)

Although *B. infantis* is a model microorganism for HMO utilization, the active metabolic pathways, fluxes, and metabolite exchange reactions associated with the metabolism of distinct HMOs molecules remain poorly understood. To address this limitation, a refined genome-scale network reconstruction of *B. infantis* metabolism (*i*LR578) was built consistent with recent literature information and transcriptomic data. The former reconstruction was then employed to generate four context-specific GSMMs describing growth on distinct carbon sources such as lactose or HMOs such as LNnT, 3′FL, and 6′SL. Our results revealed emergent patterns of metabolic adaptations of *B. infantis* under different HMOs utilization conditions.

## MATERIALS AND METHODS

### Genome-scale network reconstruction and model building

**Fig 1:**
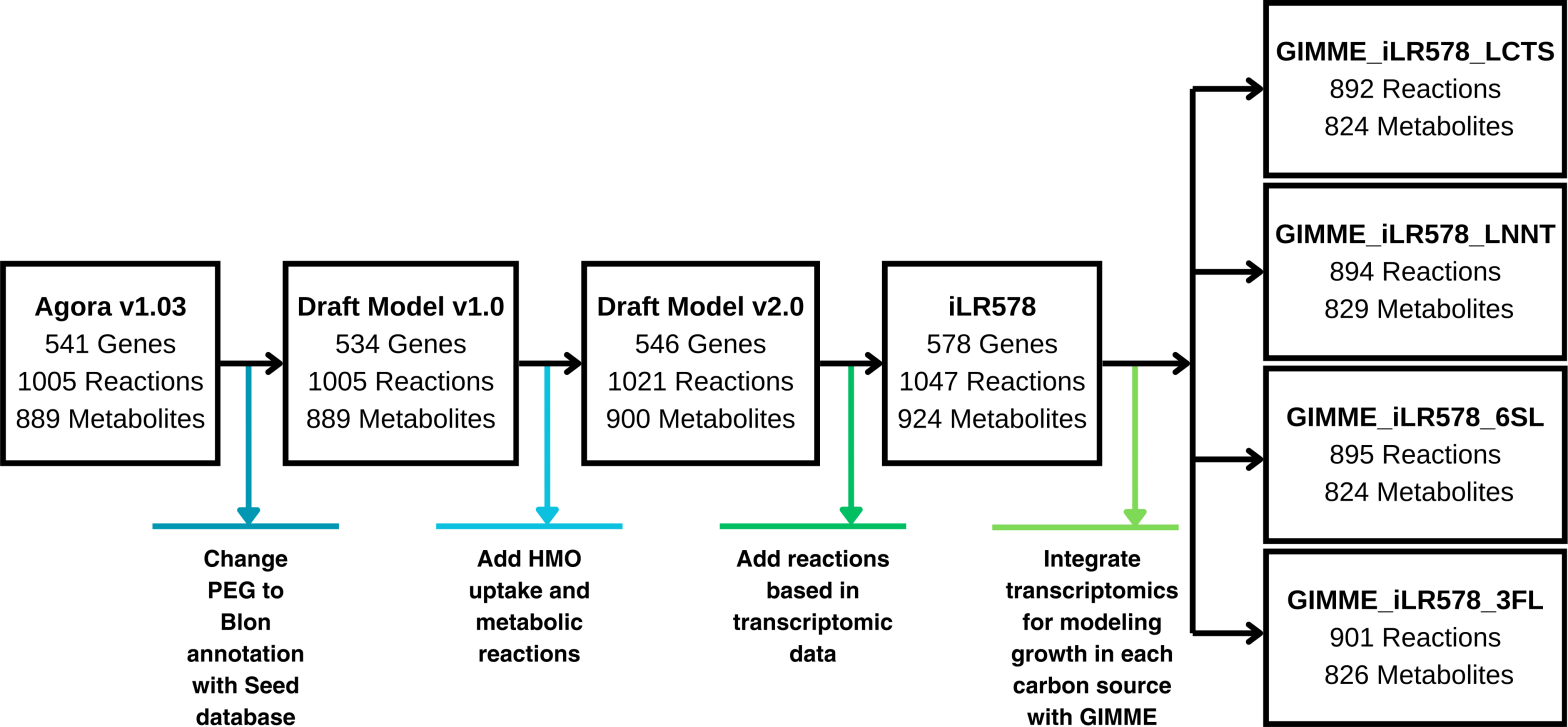
Metabolic network reconstruction and refinement workflow for building *i*LR578 and context-specific models of *B. infantis* metabolism.

Reconstruction and refinement of *B*. *infantis* (*i*LR578) metabolic network involved multiple steps (Fig. 1). First, data from multiple databases and references were employed to define gene-protein-reaction (GPR) relationships for each gene in the reconstruction. This process is used as a template for the reconstruction of Bifidobacterium_longum_infantis_ATCC_15697 from AGORA 1.03 (Assembly of Gut Organisms through Reconstruction and Analysis) (38) and integrated genome annotation information for this strain (39). Genes were manually translated from PEG to Blon annotation using the SEED database (40). Context-specific metabolic models were constructed using computational reconstruction tools from COBRA Toolbox v3.0 for MATLAB (41). Transcriptomic data from a previous study (28) were used to confirm gene expression under different carbon sources (see below). This information was integrated into each metabolic model and employed to gap-fill the model using flux balance analysis (FBA) to maximize the specific growth rate as a biological objective (42). Additional reactions and metabolites were added until the list of experimentally expressed genes was consistent with the network reconstruction in all carbon sources. Moreover, the removal of reactions and genes without experimental support or evidence against their presence in the template model was also carried out (Supplemental Data). Finally, two biomass reactions were employed to simulate growth: a generalized reaction in the original AGORA v1.03 reconstruction and a recent biomass reaction from a close strain (*B. longum*) (12).

For the initial integration of the model with transcriptomics data, normalized counts for each gene and condition were determined using a variety of preprocessing and normalization steps (see next section). Then, reactions associated with each expressed gene were evaluated for their capacity to carry flux while allowing biomass growth (Supplemental Data). The list was checked manually, and reactions and metabolites were added based on information from KEGG pathways (43) and BiGG Models (44). Finally, the quality of the metabolic reconstruction was assessed with the open tool MEMOTE (MEtabolic MOdel TEst suite) (45).

### Transcriptomics data quality control and preprocessing

Preliminary analysis of the transcriptomic data suggested a potential contamination with reads of another *Bifidobacterium* (*Bifidobacterium bifidum*). For this task, a quality control step was performed using FastQC (46) and MultiQC (47), and low-quality reads were trimmed using Trimmomatic (48) with the following parameters: leading 3, trailing 3, sliding window 3:15, and minlen 36. To eliminate *B. bifidum* reads, the transcriptomic libraries of *B. infantis* were aligned to the *B. bifidum* genome using bowtie2 (49). Subsequently, samtools was used to obtain the read identifiers. Using BBMap and Hisat2 (50, 51), reads mapped to *B. bifidum* were removed from the data set. To assess the removal effect of filtered libraries, a principal component analysis was conducted to compare the behavior of the counts data of replicates under different conditions (Fig. S1). After removing the contaminated libraries, the processed data displayed a similar behavior for all but two conditions (2'-fucosyl lactose and lacto-N-neotetraose), forming clusters at approximately the same locations. This pointed to a robust data set behavior, supporting the execution of the next steps. The preprocessed data were finally normalized using the median of ratios method from the DESeq2 (52) package to enable analysis across conditions.

### Growth simulation under different carbon sources

FBA simulations were performed to probe *B. infantis* metabolism under different carbon sources. Gurobi 9.0.1 (Beaverton, Oregon) was employed as a linear programming solver in all computations. Briefly, FBA (equation 1) solves a linear optimization problem whose objective is to maximize the flux through the biomass reaction in the model. ***S*** is the stoichiometric matrix, ***v*** is the metabolic flux vector, and *μ* ⊂ support(***v***) is the specific biomass growth rate (objective function). Upper and lower bounds for the reaction fluxes are captured by the **ub** and **lb** vectors, respectively. These vectors are defined according to each simulated condition such that they support growth in a defined *in silico* medium with distinct exchange reactions potentially active. Reactions associated with genes not expressed based on transcriptomic data have both bounds set to zero.


(1) Max. μs.t.S⋅v=0lb≤v≤ub

Typically, the linear optimization formulation in equation 1 has multiple alternative optimal flux solutions. An FBA variant known as parsimonious FBA (pFBA) (53) was employed to determine a biologically relevant flux distribution. This method is known to produce flux distributions that approximate optimal enzyme usage and avoid infeasible loops (54–56). In addition, flux variability analysis (FVA) was employed to evaluate alternative solutions by computing the flux ranges for relevant reactions (i.e., network flexibility) under (sub)optimal conditions (57).

The *in silico* medium used for all simulations was based on a modified Man Rogosa Sharp medium containing 2% (wt/vol) of each carbon source. The carbon sources were lactose and three HMOs: LNnT, 3′FL, and 6′SL. To compare yield calculation across conditions, the total number of moles for each substrate contained in 200 µL at 2% (wt/vol) was calculated and later normalized by the moles of lactose. Other carbon sources were also normalized by the moles of lactose for a fair comparison (Table S1). Similarly, amino acids, vitamins, and nucleotides were proportionally normalized and asserted that they did not limit growth. Bounds for alternative carbon sources absent in the media were set to zero.

### Evaluation of the refined metabolic model prediction capabilities

The third step was the evaluation of the prediction fidelity of the model. Simulated growth of context-specific models was validated against experimental data (28) and literature (16, 58–67). Data on growth rates, metabolite consumption, and production (10) were used to assess model predictions. Briefly, a binary analysis was performed where the experimental growth of *B. infantis* on different carbon sources was compared against *in silico* predictions. In this case, four outcomes can be obtained. A true positive (TP) indicates that both the model and data indicate growth on a particular condition; a false positive (FP) implies the model can grow on a given substrate but is not supported by the experimental evidence; a true negative (TN) indicates that both the model and data agree that no growth is observed; and, a false negative (FN) implies the model does not support growth, but experimental evidence does. The *F*-score is a standard measure of the prediction capabilities of the model (equation 2) (68)]. Additionally, Matthew’s correlation coefficient (MCC) was also calculated as it is regarded as a stricter measure of prediction power, producing a high score (MMC = 1) only if the prediction obtained good results in the outcomes above (69) (equation 3).


 (2) $$F=\frac{TP}{TP+\frac{1}{2}(FP+FN)}$$

(3) $$MCC=\frac{TP*TN-FP*FN}{\sqrt{\left(TP+FP\right)*\left(TP+FN\right)*\left(TN+FP\right)*(TN+FN)}}$$

### Transcriptomic data integration

The fourth step was integrating transcriptomics data into the model. For this task, RNA-seq data from Garrido et al. (28) for *Bifidobacterium* (particularly *B. infantis* ATCC 15697) growing exponentially on lactose and three HMOs (3′FL, 6′SL, and LNnT) were employed. The GIMME algorithm (35) was applied to build four context-specific models. Briefly, GIMME uses a user-defined expression threshold corresponding to the 30th percentile to determine if a gene has a low/high expression and then deactivates the reactions below the former. This choice is based on typical choices reported elsewhere (35) and yielded similar simulation results (Table S13). Simply put, this algorithm minimizes the usage of low-expression reactions while maximizing the biomass reaction flux (biological objective). In this way, the resulting model achieves greater consistency with the available data and assumed biological objective (35). Reactions with an “AND” GPR association are associated with the corresponding lowest expressed gene, while those with an “OR” GPR association are associated with the corresponding highest expressed gene.

### Gene essentiality analysis and flux-gene expression maps

Essential gene lists were generated by simulating single-gene deletions in all conditions. An essential gene is defined as a gene that, when knocked out, causes null growth. The corresponding reactions were identified as essential reactions. To visualize flux distribution results after FBA simulations, metabolic maps were generated using Escher (70), an online application for the integrated visualization of flux and transcriptomic data. MAT models were translated to JSON format and then uploaded to Escher, along with fluxes and a transcriptomic data table to visualize network expression maps. An integrated map for each condition was manually generated.

### Flux coupling network analysis

To reveal emergent metabolic properties under each growth condition, Flux coupling analysis (FCA) was performed using the F2C2 tool version 0.95b (71) to reveal flux dependencies (coupling) between reactions under each condition. For this task, we filtered networks encompassing major end products that contained exchanges for lactate, acetate, succinate, formate, ethanol, and 1,2-propanediol (1,2-PD) in each condition. Coupled reaction networks were identified, and their topological parameters were calculated and visualized using Cytoscape (72) as in Dal’Molin et al. (73). Finally, to visualize differences in connectivity between the four context-specific models, we built a consensus network with Dynet (74). For each node, the linkages shared between two or more networks and those unique to each network were identified. Then, changes (rewiring) were quantified using the rewiring score metric (Dn-score [74]).

## RESULTS

### Reconstruction and refinement of a GSMM for *B. infantis*

In this work, we combined metabolic modeling and transcriptomics to refine and expand the metabolism of *B. infantis* during the utilization of lactose and three HMOs (3′FL, 6′SL, and LNnT). The workflow followed is presented in Fig. 1. The initial AGORA model had 541 genes in PEG annotation, 1,005 reactions, and 889 metabolites in two different compartments (Table 1).

**TABLE 1:** Reaction content comparison for different reconstructions

Reaction features	AGORA v1.03	*i*LR578
Total number of reactions	1,005	1,047
Universally blocked reactions	279	287
Metabolic reactions	714	760
Transport reactions	133	135
Reactions with GPR associations	689	893

To integrate the genome-scale transcriptomics data into the metabolic model, the PEG annotation was manually translated to Blon annotation using the SEED database (75), yielding Draft Model v1.0 (Fig. 1). One PEG gene did not have a match in the Blon annotation, but it had a match in another *B. infantis* genome (BLIJ_2570, “pbiosynthesis”). This reaction had several genes in OR association, so it was assumed unimportant (Supplemental Data). Then, genes associated with reactions and metabolites involved in HMOs utilization were added to the reconstruction. This addition expanded the gene list to 546 genes, with 1,021 reactions and 900 metabolites (Fig. 1). Finally, GPR relationships were included for each gene based on the available experimental and reported information.

The model was later integrated with transcriptomics data. Reactions associated with expressed genes were individually checked to see if they could carry flux while ensuring biomass production under each condition. For this task, a generic GSMM of *B. infantis* was built using the current reconstruction, expression, and carbon source uptake data, thereby enabling the assessment of its growth capabilities using (p)FBA (76). In this step, 23 reactions, 24 metabolites, and 32 genes were mapped and incorporated into the model. Relevant GPRs were recovered from a previous model (12). In addition, two biomass reactions and the nongrowth-associated maintenance energy were included, although the latter was not constrained due to the lack of data. The list of blocked reactions contained 20 genes associated with 33 reactions; these were blocked in the model (i.e., carried zero flux) but are present as expression data supports them (Table S2).

The resulting metabolic model was labeled *i*LR578, a functional GSMM able to produce biomass for *B. infantis* in all the studied conditions. The latter model contained 578 genes, 1,047 reactions (893 are gene-associated), and 924 metabolites (Table 1). Metabolic reactions represented 72.6% of total reactions (760 reactions), whereas transport and exchange reactions were 135 and 149 (12.9% and 14.2% of the total number of reactions, respectively; see Table 1). While the number of blocked reactions slightly increased, the number of metabolic reactions mapped onto *i*LR578 also grew from 714 (AGORA v1.03) to 760 (6.4% net increase; Table 1). Notably, the numbers of genes, reactions, metabolites, and GPRs of *i*LR578 are higher than other related bifidobacterial GSMMs reported to date (7). For instance, the current model *B. infantis* ATCC 1,597 has 541 genes, 1,005 reactions, and 889 metabolites, whereas *B. longum subsp. longum* 157F has 519 genes, 914 reactions, and 853 metabolites. This could correlate with the larger genome size of *B. infantis* ATCC 15697 (15). Notably, while the general reconstruction indicators of *i*LR578 were improved over the AGORA v1.03 reconstruction (Table 1), the MEMOTE score—a quality metric standard for genome-scale network reconstructions—did not increase (in both cases, the score was 43, Supplementary Data), despite having an increase of 16.1% of mapped metabolites.

The MEMOTE score evaluates GSMMs compliance with community sharing and annotation standards, incorporation of relevant meta-data (e.g., annotations, GPRs, metabolite formulas, etc.), as well as different simulation checks (e.g., growth feasibility, flux cycles, and metabolic imbalances predictions, among others) (45). Yet, this suite does not cover specific metabolic capabilities under different conditions and their consistency against experimental data. Experimental evaluation of the predictive power of *i*LR578 is assessed in the next section.

### Model prediction evaluation: growth and metabolic profiles

Exponential phase growth data from a previous study using four different carbon sources (28) was employed to evaluate the predictive power of the *i*LR578, checking biomass production under anaerobic conditions using pFBA. Before this analysis, the prediction capabilities of the two biomass reactions were evaluated. Results showed that the generalized biomass reaction from AGORA was superior, reaching a 55.3% lower root-mean-square error (RMSE) when predicting the specific growth rate under the assumption of biomass maximization (mean RMSE 0.048 vs 0.108 h^−1^; Table S6). These results are somewhat to be expected, considering that there is a twofold difference in the growth-associated maintenance energy (GAM) of the two reactions (20 mmol·gDCW^−1^ vs 40 mmol·gDCW^−1^ for the Schöpping et al. and AGORA biomass reactions, respectively; Supplementary file). Besides, out of the 74 precursors of the biomass reaction reported by Schöpping et al. (12), 64 could be successfully mapped onto the *i*LR578 model (Supplemental file ). It is worth noting that the latter biomass reaction was developed for *B. longum* subsp*. longum* BB-46 growing on sucrose (12), which greatly differs from the simulation conditions of this study. In fact, even when adjusting and equating the GAM parameter, the latter equation displays a slightly higher RMSE than the AGORA v1.03 biomass reaction (2% greater; Table S6). Considering these results and the lack of additional experimental data under the studied conditions (e.g., chemostats under various dilution rates) and/or gene essential data, we decided to employ the AGORA v1.03 biomass reaction for the remaining of the study.

A theoretical ratio of 3:2 is expected for acetate to lactate in the *B. infantis* metabolism (6), so the former was imposed in the subsequent flux calculations. To assess the impact of the magnitude of the production ratio, the latter was set to vary from 2.5:2 to 3.5:2 of acetate to lactate. Results showed no statistically significant differences in the predicted specific growth rates (one-way ANOVA *P*-value = 0.971, data available in Table S7) or the maximum biomass yields (one-way ANOVA *P*-value = 0.987, data available in Table S8). Although only a modest increase (1.7%) in the predicted specific growth rate was determined when imposing a 3.5:2 ratio production of acetate to lactate (Table S7), this ratio condition will be employed in the following for the analysis.

Experimental biomass yields were similar for LNnT and approximately twice that of 3′FL and 6′SL (Table 2). Prediction of biomass yields on LNnT agreed with model predictions using pFBA. However, predictions in the remaining conditions overestimated the biomass yield except for lactose (Table 2). The production yields of major metabolic end products, such as lactate, acetate, ethanol, and succinate, were also evaluated under the assumption of biomass growth maximization (Table 3). Again, there were no substantial differences when employing different production ratios of acetate to lactate (Table S9 and S10). LNnT, 3′FL, and 6′SL are HMOs that contain lactose as a building block. Model predictions showed higher acetate and lactate yields on lactose followed by 6′SL. Overall, low formate and no succinate production were determined *in silico*. Fucose metabolism in *Bifidobacterium* uniquely results in pyruvate and 1,2-PD (64) as an end-product (Table 3). Application of flux fariability analysis for the determination of the yield ranges for the major end products under near-optimal conditions (i.e., 99% of the optimal specific growth rate) produced tight ranges, pointing to similar results and confirming the observed metabolic trends shown in Table 3 (Table S11). Most notably, even when the ratio constraint for acetate and lactate is relaxed under near-optimal conditions, the computed yield ranges are only slightly larger, and they overall display the same trends, i.e., high acetate and lactate production and very low formate and ethanol, and no succinate (Table S12).

**TABLE 2:** Experimental and predicted maximum biomass yield (10^3^⋅gdcw⋅g^−1^) by *i*LR578 under different carbon sources

Carbon source	Experimental yield^*a*^	*In silico* yield prediction from *i*LR578	*In silico* yield prediction from contextualized *i*LR578 models
Lactose	28.0	15.2	13.9
LNnT	21.3	19.0	17.9
3FL	14.8	33.6	30.3
6SL	13.1	24.7	22.2

^*a*^ Data taken from Garrido et al. (16), Garrido et al. (77), Garrido et al. (10), and Garrido et al. (28). Yields were calculated assuming a 3.5:2 acetate to lactate production ratio based on experimental observations. This ratio maximized the specific growth rate for all substrates and was therefore chosen for subsequent analyses (Table S11).

**TABLE 3:** Yield of major metabolites (g⋅gDW^−1^) predicted by *i*LR578 assuming biomass maximization under different carbon sources^*a*^

Metabolite	Carbon source			
	Lactose	LNnT	3FL	6SL
1,2-PD	0	0	0.76^*b*^	0
Acetate	2.40^*b*^	2.50	2.72	2.66
Ethanol	0.02^*b*^	0.02	0.02	0.02
Formate	0.02^*c*^	0.02	0.02	0.02
Lactate	2.10^*b*^	2.18	2.38	2.32
Succinate	0^*c*^	0	0	0

^*a*^ Evidence for metabolite production taken from Garrido et al. (10), Van der Meulen et al. (11), and Cheng et al. (78). Yields were calculated assuming a ratio of 3.5:2 acetate to lactate production ratio as in Table 2.

^*b*^ Production was observed experimentally.

^*c*^ No production was observed experimentally.

Finally, *i*LR578 showed an increase in sensitivity and *F*-score compared to the initial AGORA v1.03 model when predicting the growth of various carbon sources (Table 4; Table S4) (68). The former model had 34 TP, four FPs, and four FN. This increase was explained by *i*LR578 having more TPs and fewer FNs than the original model. Amino acid metabolism appears to be accurately represented by the metabolic model, considering *B. infantis* is only auxotrophic for L-cysteine (79). The *F-*score of 89.4% indicates that there is still space to improve the metabolic predictive capabilities of the model. *Bifidobacterium* genomes usually have many hypothetical proteins, and their metabolism is still not completely understood (15). A higher MCC value for *i*LR578 compared to the initial model corroborates the improvements made to this GSMM (Table 4).

**TABLE 4:** Performance comparison of GSMMs of *B. infantis^a^*

Performance indicator	AGORA v1.03	*i*LR578
TP	29	34
TN	6	6
FP	4	4
FN	9	4
Sensitivity	0.743	0.755
Specificity	0.555	0.555
*F*-score	0.783	0.894
MCC	0.309	0.482

^*a*^ Data available in Table S4.

### Context-specific models of HMO utilization based on transcriptomic data integration

**Fig 2:**
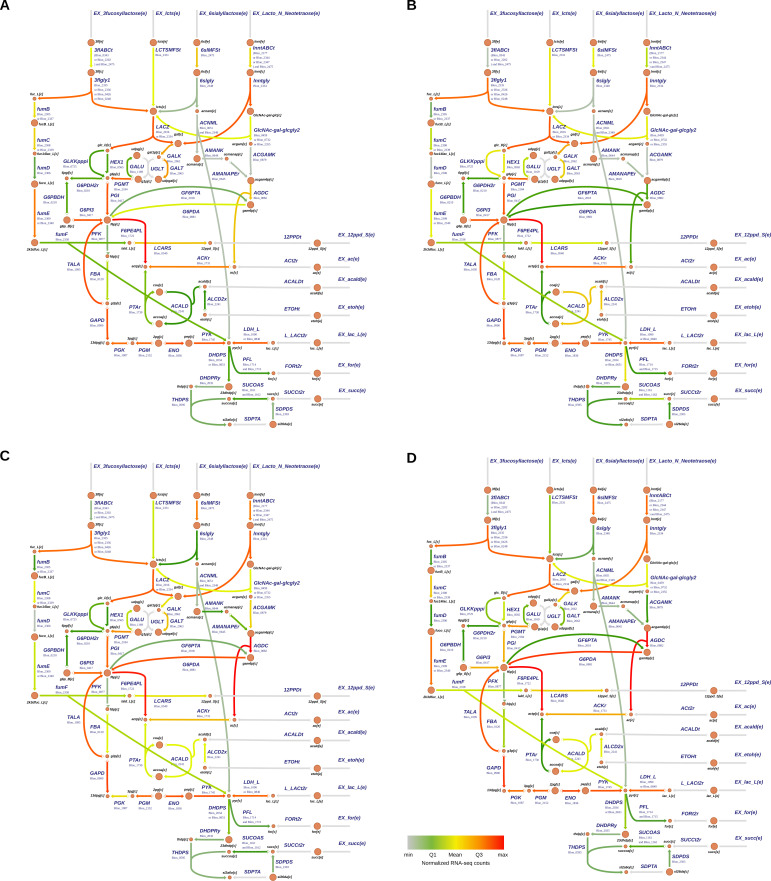
Gene expression maps of central carbon metabolism of *B. infantis* under different carbon sources. Each arrow describes a reaction, and its color intensity and width are proportional to the corresponding gene expression. Metabolic pathways depicted include substrate uptake (top), bifid shunt (middle), and major fermentation products (bottom). Panels describe growth under different carbon sources, namely, (A) 3′FL, (B) lactose, (C) 6′SL, and (D) LNnT. Metabolite nomenclature and gene symbols are found in the Supplemental Data.

Context-specific GSMMs were built from *i*LR578 and transcriptomic data on each HMO (Fig. 2). This integration resulted in four carbon source-specific models for 3′FL, lactose, 6′SL, and LNnT (Fig. 1; Supplemental Data). The highest number of deactivated transcriptionally turn-off reactions was observed in the LNnT model, with 152 reactions, followed closely by the 6′SL model (153 reactions), then the 3′FL model (145 reactions), and finally, the lactose model, with 140 deactivated reactions (Fig. 1). Context-specific models showed similar biomass yields compared to the generic *i*LR578 (Table 2).

We first analyzed gene expression patterns in these context-specific models. 3′FL is correctly imported by an ABC transporter and degraded intracellularly, generating fucose and lactose (Fig. 2A). Gene expression for genes converting lactose in galactose and glucose (including the Leloir pathway) and their conversion to glucose-6-phosphate was medium to high. Similarly, the putative fucose metabolic pathway showed medium to high expression, resulting in the production of 1,2-PD through lactaldehyde reductase (Blon_0540), acetate (Blon_1731; acetate kinase), and lactate (Blon_ 1090 or Blon_0840; lactate dehydrogenase). Therefore, this context-specific model can capture microbial growth and metabolism of *B. infantis* in 3′FL, resulting in biomass and metabolite production.

The lactose model showed similar gene expression patterns as 3′FL, without fucose metabolization and 1,2-PD production (Fig. 2B). It showed an increased gene expression for reactions at the entry of lactose through lactose permease to the bifid shunt until the production of lactate and acetate (Fig. 2B). Formate and succinate production was lower than acetate and lactate, which correlated with a lower gene expression of Blon_1714, Blon_1715 (pyruvate formate lyases, PFL), and Blon_2035 (dihydrodipicolinate synthase, DHDPS), respectively.

6′SL is imported intracellularly in *B. infantis* (18); however, the transporter is unknown. Like other HMO, 6′SL is likely imported by an ABC transporter. While gene expression supported a 6′SL-derived lactose metabolization like 3′FL or lactose, sialic acid utilization resulted in additional pyruvate and acetate production, with increased gene expression of the overlapping aminosugar metabolic pathway (Fig. 2C; Blon_0644, N-acetyl-D-mannosamine kinase; Blon_0645, N-acetylmannosamine 6-phosphate epimerase; Blon_0882, N-acetylglucosamine-6-phosphate deacetylase; Blon_0881, glucosamine-6-phosphate deaminase). However, certain enzymes in the sialic acid metabolism showed a low or null gene expression, which could indicate that other genes participate in this process, or *B. infantis* does not fully use sialic acid.

Finally, LNnT is a tetrasaccharide that, according to its model, generates intracellular galactose, lactose, and GlcNAc (Fig. 2D). According to gene expression, galactose and lactose follow similar processing steps as above. *B. infantis* β-hexosaminidases, releasing GlcNAc from LNnT, have a constitutive gene expression (Fig. 2; Blon_0459, Blon_0732, Blon_2355). Later, GlcNAc is metabolized by Blon_0879 (N-Acetylglucosamine Kinase), Blon_0881, and Blon_0882 as above.

Gene expression in the context-specific metabolic models showed several reactions with unexpected or crossed expressions. Some examples are the expression of LNnT transport and GlcNAc metabolism in the presence of 3′FL (Fig. 2A), α-fucosidases during growth in lactose, 6′SL, and LNnT (Fig. 2B through D), and some fucose metabolizing enzymes during growth in LNnT (Fig. 2D). As described previously (28), *B. infantis* is likely prepared to use multiple HMOs simultaneously, suggesting that HMOs are not usually sensed as only one molecule by *B. infantis*.

### Comparison of flux patterns and gene expression in the central metabolism of iLR578 under each carbon source

**Fig 3:**
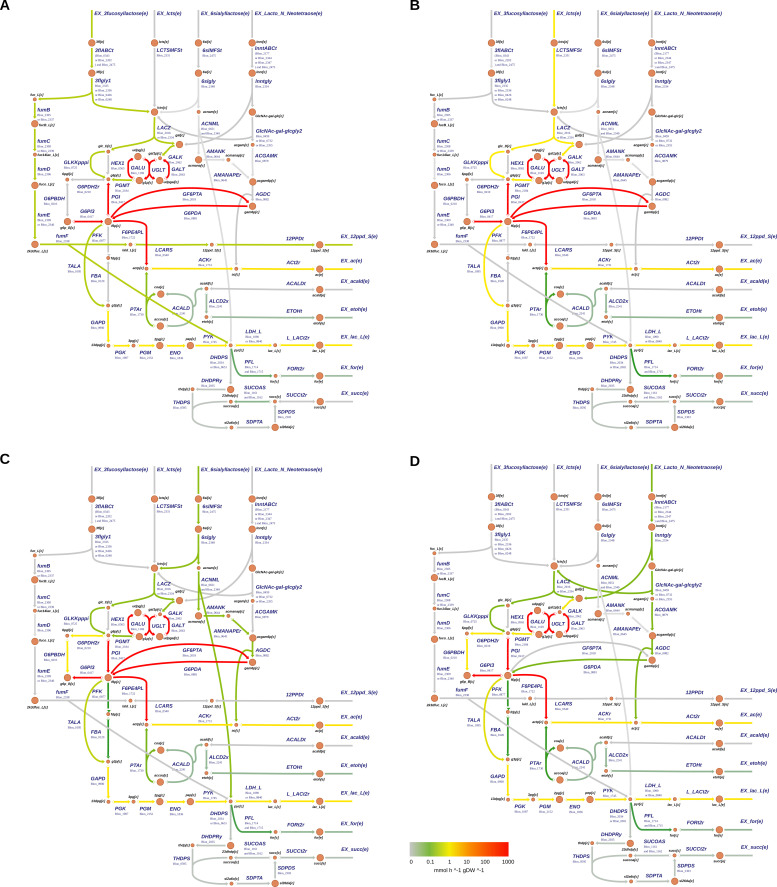
Flux distribution maps of central carbon metabolism of *B. infantis* integrating transcriptomics data. Each arrow describes a reaction, and its color intensity and width are proportional to the corresponding flux value expression. Metabolic pathways depicted include substrate uptake (top), bifid shunt (middle), and major fermentation products (bottom). Metabolic maps were generated using GIMME assuming specific growth rate maximization. Panels describe growth under different carbon sources, namely, (A) 3′FL, (B) lactose, (C) 6′SL, and (D) LNnT. Metabolite nomenclature and gene symbols are found in the Supplemental Data.

Fluxomics data showed less complex patterns than transcriptomics (Fig. 3). No crossed reactions were observed when considering metabolic fluxes. Metabolic fluxes were favored through the bifid shunt pathway in all HMOs and lactose (Fig. 3), with differences in feeder pathways according to the nature of the compound. In all four distribution maps, the carbon flux goes from sources to products, favoring the production of lactate and acetate in the previously set ratio. Reactions in the bifid shunt presented relatively high fluxes during consumption of these four substrates, which correlated with an increased gene expression (Fig. 2 and 3). They include enzymes such as fructose-6-P erythrose-4-P lyase (F6PE4PL or F6PPK), transaldolases and transketolases (TALA, TKK1, and TKK2), and PKL. The main products of the bifid shunt, acetyl-P, and GA3P were further metabolized into acetate and lactate (Fig. 3).

Figure 3B shows the flux distribution for central pathways in *B. infantis* during lactose utilization, the simplest of the substrates evaluated. Lactose is predicted to be imported by a lactose permease and hydrolyzed by a β-galactosidase. The fluxes of these reactions and galactokinase showed medium flux values (Fig. 3B). Galactose and glucose are derived into central metabolism by feeder pathways such as the Leloir pathway, which includes gene products such as UDPG4R (UDP-glucose-4-epimerase), GALU (UTP-glucose-1-phosphate uridylyltransferase), and UGLT (UDPglucose-hexose-1-phosphate uridylyltransferase). The fluxes of these intermediate reactions were predicted to be high (Fig. 3B).

Metabolic analysis indicates that 3′FL and 6′SL show an even flux distribution between fucose/sialic acid and lactose metabolism pathways (Fig. 3A and C). In these substrates, lactose metabolism fluxes were lower (green) than lactose as the sole carbon source (yellow). Flux distribution captured well the production of 1,2-PD and pyruvate in 3′FL, which was not produced in any other substrate (Fig. 3A), and how sialic acid generates glucosamine and pyruvate (Fig. 3C). Finally, LNnT overall metabolic fluxes were similar to 6′SL, probably due to NeuAc metabolism overlapping with GlcNAc (Fig. 3D).

Some reactions were active in the TCA cycle, but with a low flux, producing formate or ethanol (Fig. 3). Albeit at different levels, the models reflected well the production of lactate and acetate. The four models displayed low fluxes of formate and ethanol (Fig. 3), associated with pyruvate-formate lyase (Blon_1714 and Blon_1715) and alcohol dehydrogenase (Blon_2241), also showing low but positive fluxes. The models did not predict succinate fluxes, contrasting with the low but positive gene expression of enzymes such as succinyl-CoA synthetase (Fig. 2 and 3).

Finally, the metabolic models indicate the coupling of two reactions catalyzed by GF6PTA (glutamine-fructose-6-phosphate transaminase; Blon_2018) and G6PDA (glucosamine-6-phosphate deaminase; Blon_0881). The net result of their action is the deamination of glutamine for NH_4_^+^ release (Supplemental Data), which might indicate the models couple these reactions in the absence of ammonia. The expression of Blon_0881 was high in 6′SL and LNnT but not lactose or 3′FL, which correlates with this enzyme-generating glucosamine. Blon_2018 showed a low gene expression across all four conditions. However, metabolic modeling estimated high fluxes for these two reactions, which might be an artifact due to ammonia limitation.

**Fig 4:**
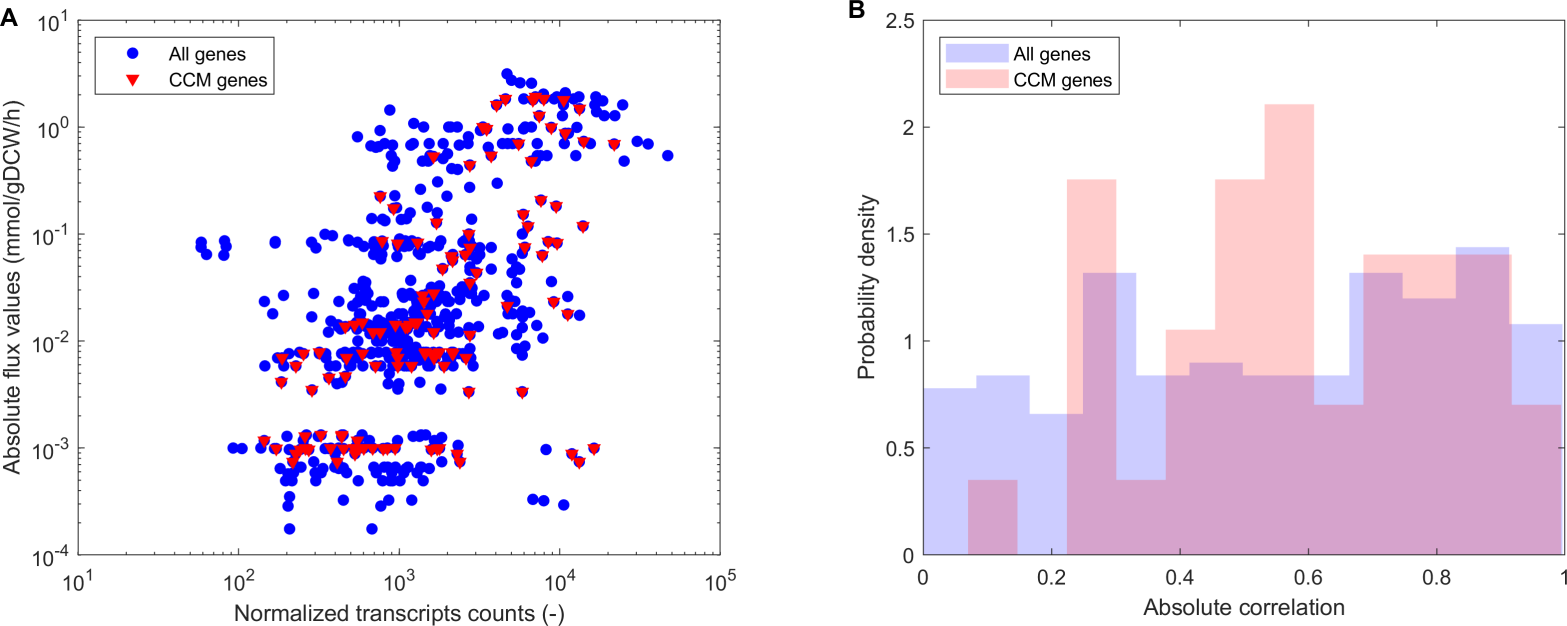
Correlation analysis between gene expression and metabolic fluxes in *B. infantis* utilizing different substrates. (A) Correlation between genes expressed consistently across all conditions and their corresponding reactions. In red, the flux and corresponding expression of central carbon metabolism genes expressed in all conditions are shown (*n* = 37), whereas in blue, the flux and corresponding expression of all genes expressed in all conditions are shown (*n* = 201). (B) Absolute correlation distribution for each consistently expressed gene and the corresponding reaction. While there is a slight increase in the mean absolute correlation of central carbon reactions and genes (0.61) vs all reactions and expressed genes (0.56), albeit this was not statistically significant (*P*-value = 0.360 > 0.05, Wilcoxon rank sum test).

The metabolic activity of almost all bifid shunt and lower glycolysis enzymes correlated with gene expression data (Fig. 4A). To assess this effect, genes expressed consistently across all conditions and their corresponding reactions were mapped, and their linear correlation coefficients were analyzed. When the absolute correlations distribution for each consistently expressed gene and the corresponding reaction was assessed, there was only a slight enrichment in the mean absolute correlation of central carbon reactions and associated genes (0.61 vs 0.56, Fig. 4B), albeit this was not statistically significant with a high confidence level (*P*-value = 0.360 > 0.05, Wilcoxon rank sum test). Overall, fluxes correlated well with gene expression, particularly in central carbon metabolism, supporting the integration of this information for gaining insights into the active metabolic pathways under each condition.

### Gene essentially simulations and analysis

Genes are predicted to be essential when their deletion results in an *in silico* growth rate of zero. The accuracy of these calculations in less studied microorganisms ranges typically between 60% and 80% (80). While there are no experimental gene lethality studies of *B. infantis* in the literature, this analysis could provide helpful information for better understanding metabolic limitations. Of the 578 genes of *i*LR578, 81 (14%) were predicted to be essential and common to all carbon sources (Table S3). As expected, genes related to critical cellular functions such as replication, translation, cargo (demand and transport reactions, 38.3%), amino acid, nucleotide, nitrogen metabolism (19.8%), and vitamins metabolism (6.2%) were most predicted to be essential (Table S5). Regardless of the carbon source, 397 genes (68.7%) did not affect biomass growth. Most of these were related to transport and demand reactions (approximately 11%).

Some genes were predicted to be nonlethal, albeit they caused a reduction in the computed growth rate. There were 76 genes of this type across the three HMOs studied (Supplemental Data). Blon_2152 (phosphoglycerate mutase) was previously predicted as an essential gene for *B. infantis* (7), and using *i*LR578, an approx. 51% reduction in growth was indicated by a single knockout. Genes predicted to be nonlethal with reduced growth rates are related to carbon source-specific pathways, ATP production, bifid shunt enzymes, and biomass-demanding metabolites (Supplemental Data).

### Identification of metabolic clusters involved in SCFAs production under different conditions

**Fig 5:**
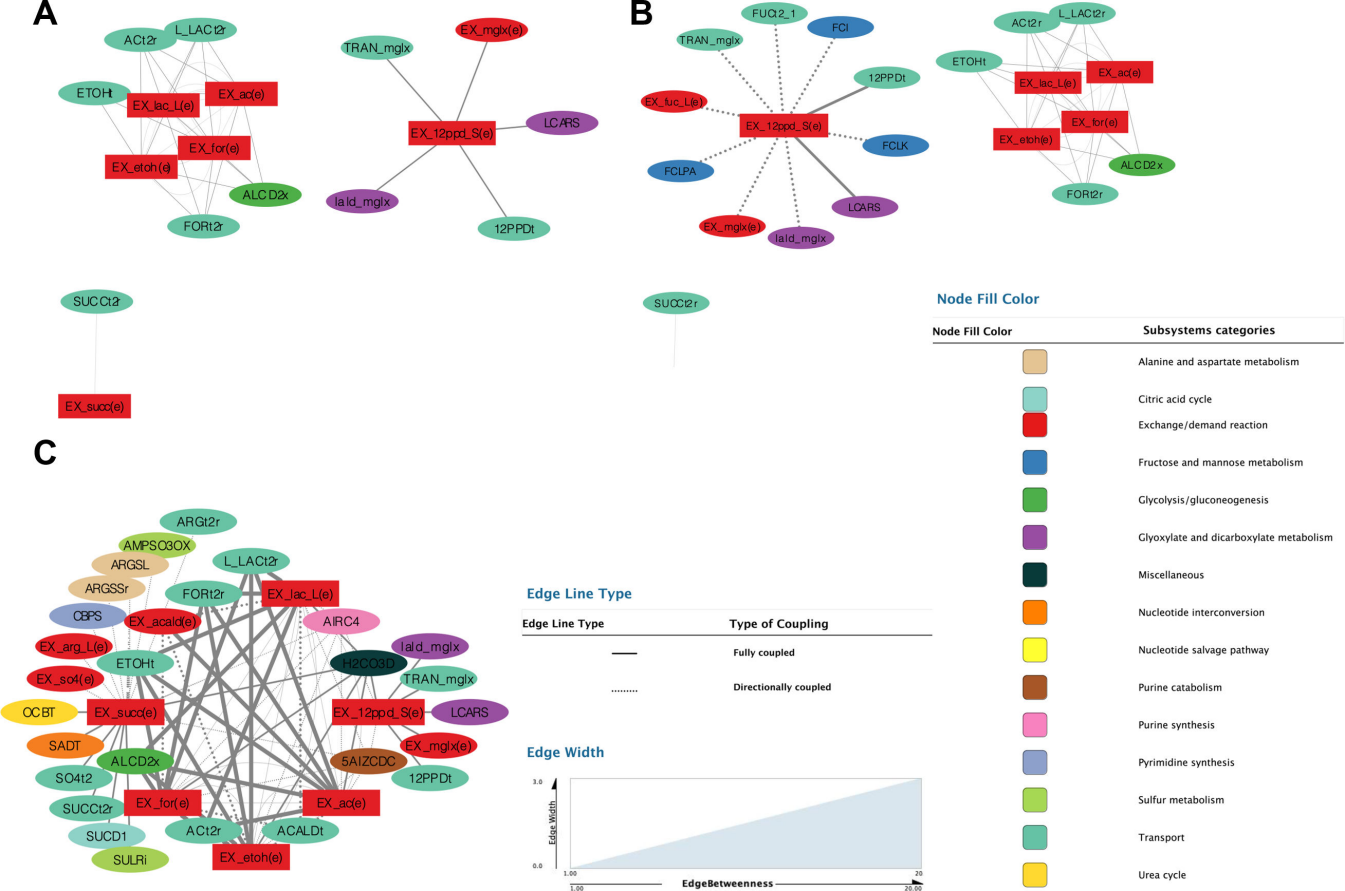
Coupled reaction networks for each context-specific model derived from FCA. For each carbon source, major fermentation metabolites, namely, lactate, acetate, succinate, formate, ethanol, and 1,2-PD (red rectangles), are depicted linked to reactions coupled to them in each condition (colored ovals). Panels describe network generated under different carbon sources, namely, (A) 3´′FL, (B) 6′SL-LNnT (identical networks), and (C) lactose. The legend on the right indicates the type of reaction coupling (partial or full—dashed or solid line, respectively), edge betweenness (line thickness), and reactions subsystems (color coded). Reaction nomenclature can be found in the Supplemental Data.

To better understand the release of major metabolites by *B. infantis* under different growth conditions, we analyzed how exchange metabolites are coupled to metabolic reactions in each context-specific model (Fig. 5). For this analysis, we again considered and imposed a 3.5:2 production ratio of acetate to lactate. The selected exchange reactions for network construction were lactate, acetate, succinate, formate, ethanol, and 1,2-PD. Connected networks of both partially and fully coupled reactions were identified (Fig. 5). Fully coupled reaction pairs are those where the increase/decrease in the flux value of one reaction causes a fixed increase/decrease in the flux of the other and vice versa (81). In the case of partially coupled reaction pairs, the extent of the increase/decrease exerted by one reaction on the other is variable and not necessarily the same in the opposite direction (81).

The network topology for 6′SL and LNnT was identical (Fig. 5). We found that the network for 3′FL was only composed of fully coupled reactions, whereas lactose, LNnT, and 6′SL had both fully and partially coupled reactions (Fig. 5). The network with the highest number of nodes was lactose, far more than the other HMOs (Table 5). Interestingly, the 3′FL network displayed the highest clustering coefficient (0.45), whereas the lactose network showed the lowest (0.29) despite having similar network sizes (Table 5). This result suggests a more coordinated—and possibly regulated—network underpinning metabolite production under LNnT than lactose. The latter was confirmed by the increased network connections (edges). Lastly, we also evaluated the effect of the acetate-to-lactate ratio on the reaction coupling and generated the reaction networks without this constraint. The resulting networks were almost identical in size and contained fewer edges as expected due to the lower number of coupled reactions (Fig. S2). Of the four networks, three maintained the number of nodes but reduced the number of edges (Fig. S2). The only exception was the LNnT network, which reduced its number of edges from 22 to 17 and now shares the same topology as the 6′SL network (Fig. S2). Notably, while the lactose network did not show a decrease in the number of nodes, its connectivity decreased substantially from 76 to 35, rendering a more disconnected network (Fig. S2). The networks remained almost identical in size and displayed similar topologies—except for lactose—suggesting the presence of specific network motifs under each condition regardless of the forcing ratio constraint.

**TABLE 5:** Topological properties of reaction networks found with FCA

Context-specific model^*a*^	Number of nodes	Number of edges	Clustering coefficient
3′FL	17	38	0.45
6′SL	22	43	0.34
LNnT	22	43	0.34
LAC	34	76	0.29

^*a*^ Reaction networks for 6SL and LNnT were identical.

In all identified networks, their degrees followed a power law (scale-free) distribution, which provides a robust network structure against random failure/perturbations (82). Lactate, acetate, ethanol, and formate are usually clustered in the same network with their corresponding transporters (Fig. 5). Interestingly, succinate exchange usually formed a separate network from other metabolites (Fig. 5).

**Fig 6:**
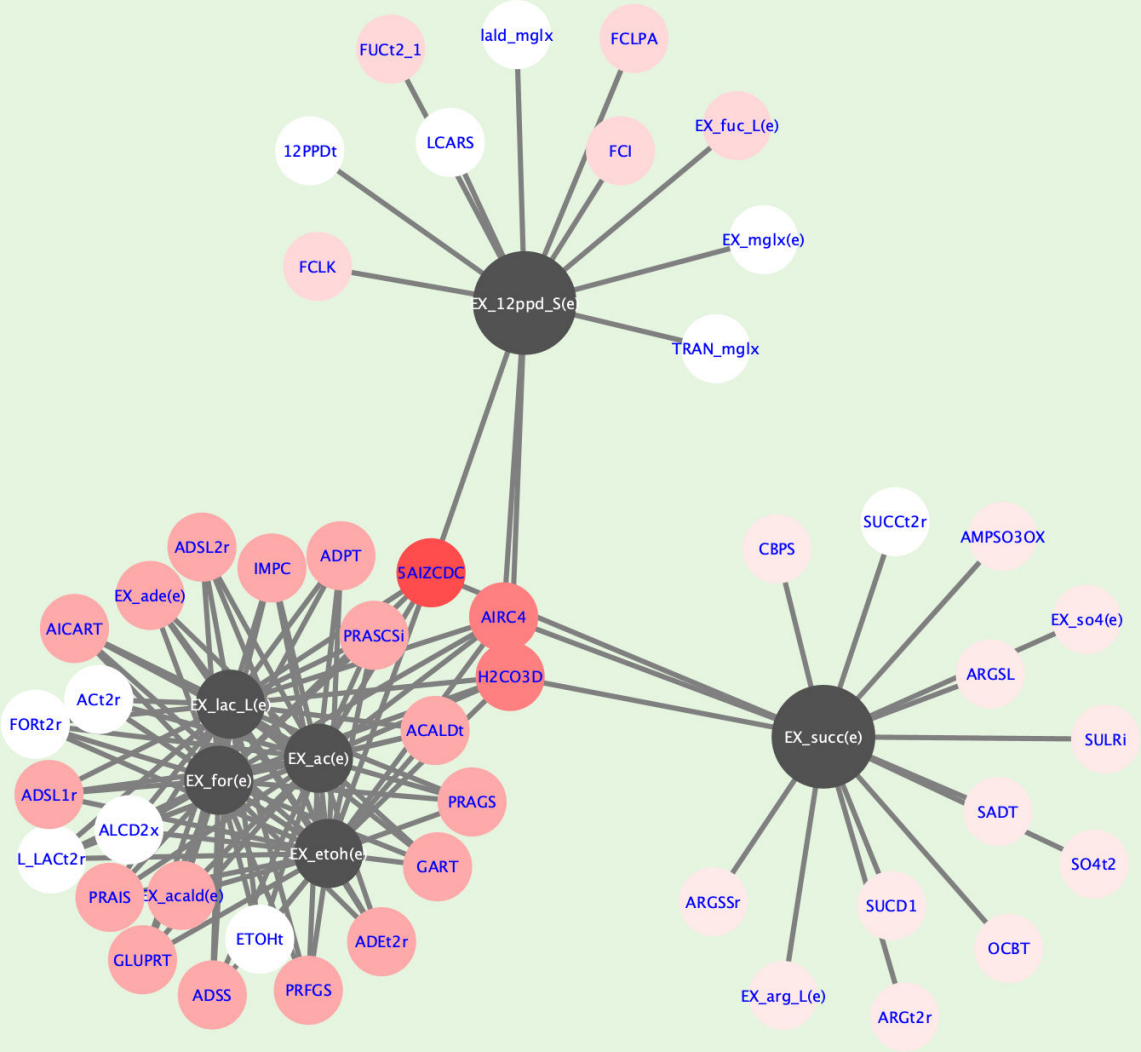
Comparison of network rewiring for building a consensus coupled reaction network. Consensus networks were constructed from all four context-specific networks using the DyNet tool. The network comprises 53 nodes and 131 edges. Black nodes represent major metabolite exchange reactions that were subject to the analysis, whereas white nodes describe reactions present in all context-specific networks. The red-shaded nodes are only present in some of the networks. Reaction nomenclature can be found in the Supplemental Data.

A global network coupling metabolites and fluxes across all four substrates is shown in Fig. 6. The Dynet analysis showed the most rewired nodes considering all four networks in Fig. 4. 1,2-PD and succinate clustered separately and were coupled to different reactions compared to lactate, acetate, formate, and ethanol (black nodes; Fig. 6). White nodes indicate reactions always coupled with these metabolites; light red nodes are reactions with a high degree of coupling. For example, lactate, acetate, formate, and ethanol appeared to be highly coupled to an alcohol dehydrogenase (ALCD2x), as well as to several reactions related to adenine and purine metabolism [ADSL, AICART, ADPT, ADSS, and EX_ade(e)]. As expected, reactions coupled to 1,2-PD were related to fucose metabolism, especially lactaldehyde reductase (LCARS). Finally, succinate was associated strongly with reactions of arginine biosynthesis, sulfate and sulfite metabolism, and succinate dehydrogenase.

## DISCUSSION

GSMMs are robust mathematical structures representing microbial metabolism, useful for understanding and optimizing metabolic pathways and microbial interactions (76). In this study, a functional GSMM of *B. infantis* was built using available transcriptomic and bibliomic information to understand HMOs utilization by this microbe. This general reconstruction served as the basis for the generation of four context-specific models describing growth on different HMOs: lactose, LNnT, 3′FL, and 6′SL. This model advances current models of *Bifidobacterium*, especially in the context of HMO utilization. A comprehensive literature search with multiple metabolites and reactions of carbohydrate consumption being used for this microorganism and the inclusion of global gene expression data helped provide a more precise and accurate representation of the consumption of HMOs by *B. infantis*.

One of the most challenging tasks for refining this model was the lack of experimental information for cellular composition, growth capabilities, and metabolite production under different media for validation. Most experimental data available for *B. infantis* are not readily applicable for model validation. GSMMs can simulate a phenotype in a specific environment. For a complex medium, approximations are employed, and care must be taken when interpreting simulation results. This adds a layer of uncertainty as the simulated production cannot be directly compared with experimental data if the simulation is deprived of some essential compound (83). Improvements in model simulations can be attained by adjusting the biomass reaction stoichiometry to experimental data such that growth and production/consumption capabilities match observations. In the case of this study, the best-performing biomass reaction was the AGORA v1.03 reaction (38), albeit the available data for testing was very limited (see Results). Condition-specific experimental data could help improve the current model’s quality, highlighting gaps in our understanding of *B. infantis* metabolism.

All *Bifidobacterium* produce lactate and acetate as part of their bifid shunt, with varying ratios depending on the substrate (6). However, their production is not well captured by metabolic models and remains an issue to improve in GSMMs. Here, the expected ratio of acetate to lactate was fixed, a solution that forces the model to produce lactate. This issue has been addressed differently in GSMMs of lactic acid bacteria that do not accurately represent lactate production (7, 33).

Compared to other reconstructions of *Bifidobacterium*, Devika et al. (7) used the AGORA model for *B. infantis* and added reactions for sialic acid and N-acetylglucosamine utilization. El-Semman et al. (37) presented a reconstruction for *Bifidobacterium adolescentis* with 454 genes and 699 reactions. Recently, Schöpping et al. (12) presented specific biomass stoichiometric equations for *Bifidobacterium animalis* and *B. longum*. The latter unfortunately did not produce satisfactory results for predicting biomass growth and yields in this study. Importantly, *i*LR578 stands as the most comprehensive genome-scale metabolic reconstruction of *B. infantis* reported to date, encompassing an increased number of genes, GPRs relations, and most importantly, metabolic capabilities.

Metabolites produced by *Bifidobacterium* are in the front line of the protective effect for the host attributed to this genus. Modifying their production might result in increased health benefits. Fucose and FL metabolism resulted in the most divergent metabolic profiles across three HMOs, strongly coupled to 1,2-PD production. The models resulting from this work predict formate being produced across all four substrates. However, experimental data suggest that formate in the infant gut derives mainly from FL and fucose metabolism (2). Finally, molecules produced at low quantities are complex to model, but it is well known that some might have a powerful effect on the host (84).

Lactate, acetate, formate, and ethanol production in *B. infantis* appeared interconnected, strongly coupling to adenine metabolism (Fig. 6). Adenine is a building block in riboflavin biosynthesis—an essential vitamin produced by *B. infantis*—suggesting HMO metabolism results in the constitutive production of riboflavin. Adenine is also related to purine metabolism, specifically DNA/RNA synthesis, which may also suggest a more direct and intuitive link to biomass biosynthesis and cellular growth. A coupling of succinate production in *B. infantis* to arginine biosynthesis was expected (6), but a connection between succinate and sulfate/sulfite metabolism deserves further investigation.

### Conclusions

*Bifidobacterium* species are essential members of the gut microbiome. Their saccharolytic lifestyle enables them to thrive on different carbon sources. *Bifidobacterium infantis* is one of the most studied microorganisms for its ability to utilize different HMOs because of their genomic and functional adaptations, including ABC transporters and saccharolytic enzymes. Modeling these adaptations using GSMMs provides an opportunity for a better understanding of the physiological features of *B. infantis* upon utilization of different HMOs, ultimately supporting the design of probiotics. In this study, we built more comprehensive and refined GSMMs of *B. infantis* metabolism, integrating context-specific transcriptomic data under three different HMOs and lactose. This reconstruction (*i*LR578) recapitulated several differences in HMOs metabolism depending on the functional characteristics of the molecules. The production of major metabolites like acetate and lactate was common to all substrates, with a small production of succinate, formate, and ethanol. Differential production of compounds like 1,2-PD was observed in the fucosylated substrate. Finally, coupled reaction networks underpinning short-chain fatty acids production were identified under each condition, providing insights into their robustness and possible regulation. Overall, *i*LR578 constitutes a valuable platform for the scientific community to simulate *B. infantis* metabolism on different HMOs, which can be further expanded and improved as more experimental data become available.

## SUPPLEMENTAL MATERIAL

Supplemental Information msystems.00715-23-s0001.docx Tables S1 to S13 and Fig. S1 and S2.

Supplemental Data msystems.00715-23-s0002.xlsx Additional data.

Legend msystems.00715-23-s0003.docx Legend for Supplemental Data.

ASM does not own the copyrights to Supplemental Material that may be linked to, or accessed through, an article. The authors have granted ASM a non-exclusive, world-wide license to publish the Supplemental Material files. Please contact the corresponding author directly for reuse.

## References

[1] Scholtens P, Oozeer R, Martin R, Amor KB, Knol J. 2012. The early settlers: intestinal microbiology in early life. Annu Rev Food Sci Technol 3:425–447. doi:10.1146/annurev-food-022811-101120 22224552

[2] Tsukuda N, Yahagi K, Hara T, Watanabe Y, Matsumoto H, Mori H, Higashi K, Tsuji H, Matsumoto S, Kurokawa K, Matsuki T. 2021. Key bacterial taxa and metabolic pathways affecting gut short-chain fatty acid profiles in early life. ISME J 15:2574–2590. doi:10.1038/s41396-021-00937-7 33723382 PMC8397723

[3] Laursen MF, Sakanaka M, von Burg N, Mörbe U, Andersen D, Moll JM, Pekmez CT, Rivollier A, Michaelsen KF, Mølgaard C, Lind MV, Dragsted LO, Katayama T, Frandsen HL, Vinggaard AM, Bahl MI, Brix S, Agace W, Licht TR, Roager HM. 2021. Bifidobacterium species associated with breastfeeding produce aromatic lactic acids in the infant gut. Nat Microbiol 6:1367–1382. doi:10.1038/s41564-021-00970-4 34675385 PMC8556157

[4] Turroni F, Milani C, Ventura M, van Sinderen D. 2022. The human gut microbiota during the initial stages of life: insights from bifidobacteria. Curr Opin Biotechnol 73:81–87. doi:10.1016/j.copbio.2021.07.012 34333445

[5] Fenster K, Freeburg B, Hollard C, Wong C, Rønhave Laursen R, Ouwehand AC. 2019. The production and delivery of probiotics: a review of a practical approach. Microorganisms 7:83. doi:10.3390/microorganisms7030083 30884906 PMC6463069

[6] Pokusaeva K, Fitzgerald GF, van Sinderen D. 2011. Carbohydrate metabolism in Bifidobacteria. Genes Nutr 6:285–306. doi:10.1007/s12263-010-0206-6 21484167 PMC3145055

[7] Devika NT, Raman K. 2019. Deciphering the metabolic capabilities of Bifidobacteria using genome-scale metabolic models. Sci Rep 9:18222. doi:10.1038/s41598-019-54696-9 31796826 PMC6890778

[8] de Vos WM, Vaughan EE. 1994. Genetics of lactose utilization in lactic acid bacteria. FEMS Microbiol Rev 15:217–237. doi:10.1111/j.1574-6976.1994.tb00136.x 7946468

[9] Walsh C, Lane JA, van Sinderen D, Hickey RM. 2022. Human milk oligosaccharide-sharing by a consortium of infant derived Bifidobacterium species. Sci Rep 12:4143. doi:10.1038/s41598-022-07904-y 35264656 PMC8907170

[10] Garrido D, Ruiz-Moyano S, Jimenez-Espinoza R, Eom H-J, Block DE, Mills DA. 2013. Utilization of galactooligosaccharides by Bifidobacterium longum subsp. infantis isolates. Food Microbiol 33:262–270. doi:10.1016/j.fm.2012.10.003 23200660 PMC3593662

[11] Van der Meulen R, Adriany T, Verbrugghe K, De Vuyst L. 2006. Kinetic analysis of bifidobacterial metabolism reveals a minor role for succinic acid in the regeneration of NAD^+^ through its growth-associated production. Appl Environ Microbiol 72:5204–5210. doi:10.1128/AEM.00146-06 16885266 PMC1538715

[12] Schöpping M, Gaspar P, Neves AR, Franzén CJ, Zeidan AA. 2021. Identifying the essential nutritional requirements of the probiotic bacteria Bifidobacterium animalis and Bifidobacterium longum through genome-scale modeling. NPJ Syst Biol Appl 7:47. doi:10.1038/s41540-021-00207-4 34887435 PMC8660834

[13] Díaz R, Torres-Miranda A, Orellana G, Garrido D. 2021. Comparative genomic analysis of novel Bifidobacterium longum subsp. longum strains reveals functional divergence in the human gut microbiota. Microorganisms 9:1906. doi:10.3390/microorganisms9091906 34576801 PMC8470182

[14] Milani C, Turroni F, Duranti S, Lugli GA, Mancabelli L, Ferrario C, van Sinderen D, Ventura M. 2016. Genomics of the genus Bifidobacterium reveals species-specific adaptation to the glycan-rich gut environment. Appl Environ Microbiol 82:980–991. doi:10.1128/AEM.03500-15 26590291 PMC4751850

[15] Sela DA, Chapman J, Adeuya A, Kim JH, Chen F, Whitehead TR, Lapidus A, Rokhsar DS, Lebrilla CB, German JB, Price NP, Richardson PM, Mills DA. 2008. The genome sequence of Bifidobacterium longum subsp. infantis reveals adaptations for milk utilization within the infant microbiome. Proc Natl Acad Sci U S A 105:18964–18969. doi:10.1073/pnas.0809584105 19033196 PMC2596198

[16] Garrido D, Kim JH, German JB, Raybould HE, Mills DA. 2011. Oligosaccharide binding proteins from Bifidobacterium longum subsp. infantis reveal a preference for host glycans. PLoS One 6:e17315. doi:10.1371/journal.pone.0017315 21423604 PMC3057974

[17] Sela DA, Garrido D, Lerno L, Wu S, Tan K, Eom H-J, Joachimiak A, Lebrilla CB, Mills DA. 2012. Bifidobacterium longum subsp. infantis ATCC 15697 α-fucosidases are active on fucosylated human milk oligosaccharides. Appl Environ Microbiol 78:795–803. doi:10.1128/AEM.06762-11 22138995 PMC3264123

[18] Thomson P, Medina DA, Garrido D. 2018. Human milk oligosaccharides and infant gut bifidobacteria: molecular strategies for their utilization. Food Microbiol 75:37–46. doi:10.1016/j.fm.2017.09.001 30056961

[19] Duranti S, Lugli GA, Milani C, James K, Mancabelli L, Turroni F, Alessandri G, Mangifesta M, Mancino W, Ossiprandi MC, Iori A, Rota C, Gargano G, Bernasconi S, Di Pierro F, van Sinderen D, Ventura M. 2019. Bifidobacterium bifidum and the infant gut microbiota: an intriguing case of microbe-host co-evolution. Environ Microbiol 21:3683–3695. doi:10.1111/1462-2920.14705 31172651

[20] Rodriguez CI, Martiny JBH. 2020. Evolutionary relationships among bifidobacteria and their hosts and environments. BMC Genomics 21:26. doi:10.1186/s12864-019-6435-1 31914919 PMC6950798

[21] Soyyılmaz B, Mikš MH, Röhrig CH, Matwiejuk M, Meszaros-Matwiejuk A, Vigsnæs LK. 2021. The mean of milk: a review of human milk oligosaccharide concentrations throughout lactation. Nutrients 13:2737. doi:10.3390/nu13082737 34444897 PMC8398195

[22] Totten SM, Zivkovic AM, Wu S, Ngyuen U, Freeman SL, Ruhaak LR, Darboe MK, German JB, Prentice AM, Lebrilla CB. 2012. Comprehensive profiles of human milk oligosaccharides yield highly sensitive and specific markers for determining secretor status in lactating mothers. J Proteome Res 11:6124–6133. doi:10.1021/pr300769g 23140396

[23] German JB, Lebrilla C, Mills DA. 2022. Milk: a scientific model for diet and health research in the 21st century. Front Nutr 9:922907. doi:10.3389/fnut.2022.922907 35757260 PMC9226620

[24] Garrido D, Ruiz-Moyano S, Kirmiz N, Davis JC, Totten SM, Lemay DG, Ugalde JA, German JB, Lebrilla CB, Mills DA. 2016. A novel gene cluster allows preferential utilization of fucosylated milk oligosaccharides in Bifidobacterium longum subsp. longum SC596. Sci Rep 6:35045. doi:10.1038/srep35045 27756904 PMC5069460

[25] Turroni F, Bottacini F, Foroni E, Mulder I, Kim J-H, Zomer A, Sánchez B, Bidossi A, Ferrarini A, Giubellini V, Delledonne M, Henrissat B, Coutinho P, Oggioni M, Fitzgerald GF, Mills D, Margolles A, Kelly D, van Sinderen D, Ventura M. 2010. Genome analysis of Bifidobacterium bifidum PRL2010 reveals metabolic pathways for host-derived glycan foraging. Proc Natl Acad Sci U S A 107:19514–19519. doi:10.1073/pnas.1011100107 20974960 PMC2984195

[26] Martin AJM, Serebrinsky-Duek K, Riquelme E, Saa PA, Garrido D. 2023. Microbial interactions and the homeostasis of the gut microbiome: the role of Bifidobacterium. Microbiome Res Rep 2:17. doi:10.20517/mrr.2023.10 38046822 PMC10688804

[27] Masi AC, Stewart CJ. 2022. Untangling human milk oligosaccharides and infant gut microbiome. iScience 25:103542. doi:10.1016/j.isci.2021.103542 34950861 PMC8671521

[28] Garrido D, Ruiz-Moyano S, Lemay DG, Sela DA, German JB, Mills DA. 2015. Comparative transcriptomics reveals key differences in the response to milk oligosaccharides of infant gut-associated bifidobacteria. Sci Rep 5:13517. doi:10.1038/srep13517 26337101 PMC4559671

[29] Ruiz-Moyano S, Totten SM, Garrido DA, Smilowitz JT, German JB, Lebrilla CB, Mills DA. 2013. Variation in consumption of human milk oligosaccharides by infant gut-associated strains of bifidobacterium breve. Appl Environ Microbiol 79:6040–6049. doi:10.1128/AEM.01843-13 23892749 PMC3811376

[30] Duar RM, Casaburi G, Mitchell RD, Scofield LNC, Ortega Ramirez CA, Barile D, Henrick BM, Frese SA. 2020. Comparative genome analysis of Bifidobacterium longum subsp. infantis strains reveals variation in human milk oligosaccharide utilization genes among commercial probiotics. Nutrients 12:3247. doi:10.3390/nu12113247 33114073 PMC7690671

[31] Li M, Zhou X, Stanton C, Ross RP, Zhao J, Zhang H, Yang B, Chen W. 2021. Comparative genomics analyses reveal the differences between B. longum subsp. infantis and B. longum subsp. longum in carbohydrate utilisation, CRISPR-Cas systems and bacteriocin operons. Microorganisms 9:1713. doi:10.3390/microorganisms9081713 34442792 PMC8399906

[32] Sakanaka M, Hansen ME, Gotoh A, Katoh T, Yoshida K, Odamaki T, Yachi H, Sugiyama Y, Kurihara S, Hirose J, Urashima T, Xiao J-Z, Kitaoka M, Fukiya S, Yokota A, Lo Leggio L, Abou Hachem M, Katayama T. 2019. Evolutionary adaptation in fucosyllactose uptake systems supports bifidobacteria-infant symbiosis. Sci Adv 5:eaaw7696. doi:10.1126/sciadv.aaw7696 31489370 PMC6713505

[33] Sen P, Orešič M. 2019. Metabolic modeling of human gut microbiota on a genome scale: an overview. Metabolites 9:22. doi:10.3390/metabo9020022 30695998 PMC6410263

[34] Altamirano Á, Saa PA, Garrido D. 2020. Inferring composition and function of the human gut microbiome in time and space: a review of genome-scale metabolic modelling tools. Comput Struct Biotechnol J 18:3897–3904. doi:10.1016/j.csbj.2020.11.035 33335687 PMC7719866

[35] Becker SA, Palsson BO. 2008. Context-specific metabolic networks are consistent with experiments. PLoS Comput Biol 4:e1000082. doi:10.1371/journal.pcbi.1000082 18483554 PMC2366062

[36] Machado D, Herrgård M. 2014. Systematic evaluation of methods for integration of transcriptomic data into constraint-based models of metabolism. PLOS Comput Biol 10:e1003580. doi:10.1371/journal.pcbi.1003580 24762745 PMC3998872

[37] El-Semman IE, Karlsson FH, Shoaie S, Nookaew I, Soliman TH, Nielsen J. 2014. Genome-scale metabolic reconstructions of Bifidobacterium adolescentis L2-32 and Faecalibacterium prausnitzii A2-165 and their interaction. BMC Syst Biol 8:41. doi:10.1186/1752-0509-8-41 24708835 PMC4108055

[38] Magnúsdóttir S, Heinken A, Kutt L, Ravcheev DA, Bauer E, Noronha A, Greenhalgh K, Jäger C, Baginska J, Wilmes P, Fleming RMT, Thiele I. 2017. Generation of genome-scale metabolic reconstructions for 773 members of the human gut microbiota. Nat Biotechnol 35:81–89. doi:10.1038/nbt.3703 27893703

[39] Chen I-M, Chu K, Palaniappan K, Ratner A, Huang J, Huntemann M, Hajek P, Ritter S, Varghese N, Seshadri R, Roux S, Woyke T, Eloe-Fadrosh EA, Ivanova NN, Kyrpides NC. 2021. The IMG/M data management and analysis system v.6.0: new tools and advanced capabilities. Nucleic Acids Res 49:D751–D763. doi:10.1093/nar/gkaa939 33119741 PMC7778900

[40] Disz T, Akhter S, Cuevas D, Olson R, Overbeek R, Vonstein V, Stevens R, Edwards RA. 2010. Accessing the SEED genome databases via web services API: tools for programmers. BMC Bioinformatics 11:319. doi:10.1186/1471-2105-11-319 20546611 PMC2900279

[41] Heirendt L, Arreckx S, Pfau T, Mendoza SN, Richelle A, Heinken A, Haraldsdóttir HS, Wachowiak J, Keating SM, Vlasov V, et al.. 2019. Creation and analysis of biochemical constraint-based models using the COBRA Toolbox v.3.0. Nat Protoc 14:639–702. doi:10.1038/s41596-018-0098-2 30787451 PMC6635304

[42] Orth JD, Thiele I, Palsson BØ. 2010. What is flux balance analysis Nat Biotechnol 28:245–248. doi:10.1038/nbt.1614 20212490 PMC3108565

[43] Kanehisa M, Sato Y, Kawashima M, Furumichi M, Tanabe M. 2016. KEGG as a reference resource for gene and protein annotation. Nucleic Acids Res 44:D457–D462. doi:10.1093/nar/gkv1070 26476454 PMC4702792

[44] King ZA, Lu J, Dräger A, Miller P, Federowicz S, Lerman JA, Ebrahim A, Palsson BO, Lewis NE. 2016. BiGG Models: a platform for integrating, standardizing and sharing genome-scale models. Nucleic Acids Res 44:D515–D522. doi:10.1093/nar/gkv1049 26476456 PMC4702785

[45] Lieven C, Beber ME, Olivier BG, Bergmann FT, Ataman M, Babaei P, Bartell JA, Blank LM, Chauhan S, Correia K, et al.. 2020. MEMOTE for standardized genome-scale metabolic model testing. Nat Biotechnol 38:272–276. doi:10.1038/s41587-020-0446-y 32123384 PMC7082222

[46] Andrews S. 2010. FastQC: a quality control tool for high throughput sequence data

[47] Ewels P, Magnusson M, Lundin S, Käller M. 2016. MultiQC: summarize analysis results for multiple tools and samples in a single report. Bioinformatics 32:3047–3048. doi:10.1093/bioinformatics/btw354 27312411 PMC5039924

[48] Bolger AM, Lohse M, Usadel B. 2014. Trimmomatic: a flexible trimmer for Illumina sequence data. Bioinformatics 30:2114–2120. doi:10.1093/bioinformatics/btu170 24695404 PMC4103590

[49] Langmead B, Salzberg SL. 2012. Fast gapped-read alignment with Bowtie 2. Nat Methods 9:357–359. doi:10.1038/nmeth.1923 22388286 PMC3322381

[50] Bushnell B. 2014. BBMap: a fast, accurate, splice-aware aligner (No. LBNL-7065E). Berkeley, CA (United States) Lawrence Berkeley National Lab.(LBNL)

[51] Kim D, Paggi JM, Park C, Bennett C, Salzberg SL. 2019. Graph-based genome alignment and genotyping with HISAT2 and HISAT-genotype. Nat Biotechnol 37:907–915. doi:10.1038/s41587-019-0201-4 31375807 PMC7605509

[52] Love MI, Huber W, Anders S. 2014. Moderated estimation of fold change and dispersion for RNA-seq data with DESeq2. Genome Biol 15:550. doi:10.1186/s13059-014-0550-8 25516281 PMC4302049

[53] Lewis NE, Hixson KK, Conrad TM, Lerman JA, Charusanti P, Polpitiya AD, Adkins JN, Schramm G, Purvine SO, Lopez-Ferrer D, Weitz KK, Eils R, König R, Smith RD, Palsson BØ. 2010. Omic data from evolved E. coli are consistent with computed optimal growth from genome-scale models. Mol Syst Biol 6:390. doi:10.1038/msb.2010.47 20664636 PMC2925526

[54] De Martino D, Capuani F, Mori M, De Martino A, Marinari E. 2013. Counting and correcting thermodynamically infeasible flux cycles in genome-scale metabolic networks. Metabolites 3:946–966. doi:10.3390/metabo3040946 24958259 PMC3937828

[55] Saa PA, Nielsen LK. 2016. Fast-SNP: a fast matrix pre-processing algorithm for efficient loopless flux optimization of metabolic models. Bioinformatics 32:3807–3814. doi:10.1093/bioinformatics/btw555 27559155 PMC5167067

[56] Saa PA, Nielsen LK. 2016. ll-ACHRB: a scalable algorithm for sampling the feasible solution space of metabolic networks. Bioinformatics 32:2330–2337. doi:10.1093/bioinformatics/btw132 27153696

[57] Mahadevan R, Schilling CH. 2003. The effects of alternate optimal solutions in constraint-based genome-scale metabolic models. Metab Eng 5:264–276. doi:10.1016/j.ymben.2003.09.002 14642354

[58] Bunesova V, Lacroix C, Schwab C. 2016. Fucosyllactose and L-fucose utilization of infant Bifidobacterium longum and Bifidobacterium kashiwanohense. BMC Microbiol 16:248. doi:10.1186/s12866-016-0867-4 27782805 PMC5080750

[59] Corona L, Lussu A, Bosco A, Pintus R, Cesare Marincola F, Fanos V, Dessì A. 2021. Human milk oligosaccharides: a comprehensive review towards metabolomics. Children (Basel) 8:804. doi:10.3390/children8090804 34572236 PMC8465502

[60] Dedon LR, Özcan E, Rani A, Sela DA. 2020. Bifidobacterium infantis metabolizes 2′fucosyllactose-derived and free fucose through a common catabolic pathway resulting in 1,2-propanediol secretion. Front Nutr 7:583397. doi:10.3389/fnut.2020.583397 33330584 PMC7732495

[61] Garrido D, Ruiz-Moyano S, Mills DA. 2012. Release and utilization of N-acetyl-d-glucosamine from human milk oligosaccharides by Bifidobacterium longum subsp. infantis. Anaerobe 18:430–435. doi:10.1016/j.anaerobe.2012.04.012 22579845 PMC7568402

[62] Kim J-H, An HJ, Garrido D, German JB, Lebrilla CB, Mills DA, de Crécy-Lagard V. 2013. Proteomic analysis of Bifidobacterium longum subsp. infantis reveals the metabolic insight on consumption of prebiotics and host glycans. PLoS ONE 8:e57535. doi:10.1371/journal.pone.0057535 23469017 PMC3582569

[63] Lugli GA, Mancino W, Milani C, Duranti S, Turroni F, van Sinderen D, Ventura M, McBain AJ. 2018. Reconstruction of the bifidobacterial pan-secretome reveals the network of extracellular interactions between bifidobacteria and the infant gut. Appl Environ Microbiol 84:1–12. doi:10.1128/AEM.00796-18 PMC6070755 29884754

[64] Ojima MN, Asao Y, Nakajima A, Katoh T, Kitaoka M, Gotoh A, Hirose J, Urashima T, Fukiya S, Yokota A, Abou Hachem M, Sakanaka M, Katayama T. 2022. Diversification of a fucosyllactose transporter within the genus Bifidobacterium. Appl Environ Microbiol 88:e0143721. doi:10.1128/AEM.01437-21 34731055 PMC8788664

[65] Özcan E, Sela DA. 2018. Inefficient metabolism of the human milk oligosaccharides lacto-N-tetraose and lacto-N-neotetraose shifts Bifidobacterium longum subsp. infantis physiology. Front. Nutr 5:46. doi:10.3389/fnut.2018.00046 29900174 PMC5989456

[66] Zabel BE, Gerdes S, Evans KC, Nedveck D, Singles SK, Volk B, Budinoff C. 2020. Strain-specific strategies of 2′-fucosyllactose, 3-fucosyllactose, and difucosyllactose assimilation by Bifidobacterium longum subsp. infantis Bi-26 and ATCC 15697. Sci Rep 10:15919. doi:10.1038/s41598-020-72792-z 32985563 PMC7522266

[67] Zabel B, Yde CC, Roos P, Marcussen J, Jensen HM, Salli K, Hirvonen J, Ouwehand AC, Morovic W. 2019. Novel genes and metabolite trends in Bifidobacterium longum subsp. infantis Bi-26 metabolism of human milk oligosaccharide 2′-fucosyllactose. Sci Rep 9:7983. doi:10.1038/s41598-019-43780-9 31138818 PMC6538704

[68] Mendoza SN, Olivier BG, Molenaar D, Teusink B. 2019. A systematic assessment of current genome-scale metabolic reconstruction tools. Genome Biol 20:158. doi:10.1186/s13059-019-1769-1 31391098 PMC6685185

[69] Chicco D, Tötsch N, Jurman G. 2021. The Matthews correlation coefficient (MCC) is more reliable than balanced accuracy, bookmaker informedness, and markedness in two-class confusion matrix evaluation. BioData Min 14:13. doi:10.1186/s13040-021-00244-z 33541410 PMC7863449

[70] King ZA, Dräger A, Ebrahim A, Sonnenschein N, Lewis NE, Palsson BO. 2015. Escher: a web application for building, sharing, and embedding data-rich visualizations of biological pathways. PLoS Comput Biol 11:e1004321. doi:10.1371/journal.pcbi.1004321 26313928 PMC4552468

[71] Larhlimi A, David L, Selbig J, Bockmayr A. 2012. F2C2: a fast tool for the computation of flux coupling in genome-scale metabolic networks. BMC Bioinformatics 13:57. doi:10.1186/1471-2105-13-57 22524245 PMC3515416

[72] Shannon P, Markiel A, Ozier O, Baliga NS, Wang JT, Ramage D, Amin N, Schwikowski B, Ideker T. 2003. Cytoscape:a software environment for integrated models of biomolecular interaction networks. Genome Res 13:2498–2504. doi:10.1101/gr.1239303 14597658 PMC403769

[73] Gomes de Oliveira Dal’Molin C, Quek L-E, Saa PA, Nielsen LK. 2015. A multi-tissue genome-scale metabolic modeling framework for the analysis of whole plant systems. Front Plant Sci 6:4. doi:10.3389/fpls.2015.00004 25657653 PMC4302846

[74] Goenawan IH, Bryan K, Lynn DJ. 2016. DyNet: visualization and analysis of dynamic molecular interaction networks. Bioinformatics 32:2713–2715. doi:10.1093/bioinformatics/btw187 27153624 PMC5013899

[75] Overbeek R, Olson R, Pusch GD, Olsen GJ, Davis JJ, Disz T, Edwards RA, Gerdes S, Parrello B, Shukla M, Vonstein V, Wattam AR, Xia F, Stevens R. 2014. The SEED and the Rapid Annotation of microbial genomes using Subsystems Technology (RAST). Nucleic Acids Res 42:D206–14. doi:10.1093/nar/gkt1226 24293654 PMC3965101

[76] Saa P, Urrutia A, Silva-Andrade C, Martín AJ, Garrido D. 2022. Modeling approaches for probing cross-feeding interactions in the human gut microbiome. Comput Struct Biotechnol J 20:79–89. doi:10.1016/j.csbj.2021.12.006 34976313 PMC8685919

[77] Garrido D, Dallas DC, Mills DA. 2013. Consumption of human milk glycoconjugates by infant-associated bifidobacteria: mechanisms and implications. Microbiology (Reading) 159:649–664. doi:10.1099/mic.0.064113-0 23460033 PMC4083661

[78] Cheng L, Kiewiet MBG, Logtenberg MJ, Groeneveld A, Nauta A, Schols HA, Walvoort MTC, Harmsen HJM, de Vos P. 2020. Effects of different human milk oligosaccharides on growth of Bifidobacteria in monoculture and co-culture with Faecalibacterium prausnitzii. Front Microbiol 11:569700. doi:10.3389/fmicb.2020.569700 33193162 PMC7662573

[79] You X, Lyu Y, Sela DA. 2016. The participation of an infant harbored bacterial commensal in urea nitrogen salvaging. The FASEB Journal 30:673. doi:10.1096/fasebj.30.1_supplement.673.3

[80] Metris A, Reuter M, Gaskin DJH, Baranyi J, van Vliet AHM. 2011. In vivo and in silico determination of essential genes of Campylobacter jejuni. BMC Genomics 12:535. doi:10.1186/1471-2164-12-535 22044676 PMC3229698

[81] Burgard AP, Nikolaev EV, Schilling CH, Maranas CD. 2004. Flux coupling analysis of genome-scale metabolic network reconstructions. Genome Res 14:301–312. doi:10.1101/gr.1926504 14718379 PMC327106

[82] Wu J, Tan S-Y, Liu Z, Tan Y-J, Lu X. 2017. Enhancing structural robustness of scale-free networks by information disturbance. Sci Rep 7:7559. doi:10.1038/s41598-017-07878-2 28790416 PMC5548747

[83] Bernstein DB, Sulheim S, Almaas E, Segrè D. 2021. Addressing uncertainty in genome-scale metabolic model reconstruction and analysis. Genome Biol 22:64. doi:10.1186/s13059-021-02289-z 33602294 PMC7890832

[84] Henrick BM, Rodriguez L, Lakshmikanth T, Pou C, Henckel E, Arzoomand A, Olin A, Wang J, Mikes J, Tan Z, et al.. 2021. Bifidobacteria-mediated immune system imprinting early in life. Cell 184:3884–3898. doi:10.1016/j.cell.2021.05.030 34143954

