# Supplementary material for: Genome-scale metabolic modeling of the human milk oligosaccharide utilization by Bifidobacterium longum subsp. infantis
Source: mSystems. 2024 Feb 16;9(3):e00715-23. doi: 10.1128/msystems.00715-23 (PMC10949479; doi:10.1128/msystems.00715-23)
Supplement: Supplemental Information — Tables S1 to S13 and Fig. S1 and S2. [file msystems.00715-23-s0001.docx]

**Supplementary Information**

# **Genome-scale metabolic modeling of the human milk oligosaccharide utilization by *Bifidobacterium longum* subsp. *infantis***

Loreto Román, Felipe Melis-Arcos, Tomás Pröschle, Pedro A. Saa, Daniel Garrido

**Supplementary Tables**

**Table S1**. Metabolites present in simulated modified Lactobacilli MRS

| **Component** | **g/L** | **g/200 uL** | **mol/200 uL** | **Normalized flux** |
| --- | --- | --- | --- | --- |
| Lactose | 20 | 4.0E-03 | 1.17E-05 | 1.0E+00 |
| Lacto-N-tetraose | 20 | 4.0E-03 | 5.65E-06 | 4.8E-01 |
| Lacto-N-neotetraose | 20 | 4.0E-03 | 5.65E-06 | 4.8E-01 |
| 2-Fucosyllactose | 20 | 4.0E-03 | 8.19E-06 | 7.0E-01 |
| 3-Fucosyllactose | 20 | 4.0E-03 | 8.19E-06 | 7.0E-01 |
| 6-Sialyllactose | 20 | 4.0E-03 | 6.31E-06 | 5.4E-01 |
| Urea | 0.5 | 1.0E-04 | 1.66E-06 | 1.4E-01 |
| GABA | 0.1 | 2.0E-05 | 1.94E-07 | 1.7E-02 |
| Alanine | 0.2 | 4.0E-05 | 4.49E-07 | 3.8E-02 |
| Arginine | 0.2 | 4.0E-05 | 2.30E-07 | 2.0E-02 |
| Asparagine | 0.1 | 2.0E-05 | 1.51E-07 | 1.3E-02 |
| Aspartic acid | 0.3 | 6.0E-05 | 4.51E-07 | 3.9E-02 |
| Cysteine | 0.6 | 1.2E-04 | 9.90E-07 | 8.5E-02 |
| Glutamic acid | 0.3 | 6.0E-05 | 4.08E-07 | 3.5E-02 |
| Glycine | 0.2 | 4.0E-05 | 5.33E-07 | 4.6E-02 |
| Histidine | 0.2 | 4.0E-05 | 2.09E-07 | 1.8E-02 |
| Isoleucine | 0.1 | 2.0E-05 | 1.52E-07 | 1.3E-02 |
| Leucine | 0.2 | 4.0E-05 | 3.05E-07 | 2.6E-02 |
| Lysine | 0.2 | 4.0E-05 | 2.19E-07 | 1.9E-02 |
| Methionine | 0.1 | 2.0E-05 | 1.34E-07 | 1.1E-02 |
| Phenylalanine | 0.1 | 2.0E-05 | 1.21E-07 | 1.0E-02 |
| Proline | 0.2 | 4.0E-05 | 3.48E-07 | 3.0E-02 |
| Serine | 0.1 | 2.0E-05 | 1.90E-07 | 1.6E-02 |
| Threonine | 0.1 | 2.0E-05 | 1.68E-07 | 1.4E-02 |
| Tryptophan | 0.1 | 2.0E-05 | 9.79E-08 | 8.4E-03 |
| Tyrosine | 0.1 | 2.0E-05 | 1.10E-07 | 9.4E-03 |
| Valine | 0.1 | 2.0E-05 | 1.71E-07 | 1.5E-02 |
| Aminobenzoic | 0.0002 | 4.0E-08 | 2.92E-10 | 2.5E-05 |
| Biotin | 0.001 | 2.0E-07 | 8.19E-10 | 7.0E-05 |
| Cyanocobalamin | 0.00002 | 4.0E-09 | 2.95E-12 | 2.5E-07 |
| Folic acid | 0.0002 | 4.0E-08 | 9.06E-11 | 7.8E-06 |
| Myo inositol | 0.01 | 2.0E-06 | 1.11E-08 | 9.5E-04 |
| Nicotinic acid | 0.01 | 2.0E-06 | 1.62E-08 | 1.4E-03 |
| Pantothenate | 0.01 | 2.0E-06 | 4.20E-09 | 3.6E-04 |
| Pyridoxal | 0.01 | 2.0E-06 | 9.82E-09 | 8.4E-04 |
| Riboflavin | 0.01 | 2.0E-06 | 5.31E-09 | 4.5E-04 |
| Cytidine | 0.1 | 2.0E-05 | 8.29E-08 | 7.1E-03 |
| Adenosine | 0.1 | 2.0E-05 | 7.96E-08 | 6.8E-03 |
| Uridine | 0.1 | 2.0E-05 | 8.76E-08 | 7.5E-03 |
| Guanine | 0.1 | 2.0E-05 | 1.32E-07 | 1.1E-02 |

**Table S2.** Reactions that should be expressed based on transcriptomics data

| **Gene** | **Reaction ID** | **Reaction Name** |
| --- | --- | --- |
| Blon_0029 | FERO | Ferroxidase |
| Blon_0070 | GLCP3 | Glycogen phosphorylase |
| Blon_0083 | ADNCYC | Adenylate Cyclase |
| Blon_0116 | FRDOr | Ferredoxin oxidoreductase |
| Blon_0794 | HMPK1 | Hydroxymethylpyrimidine kinase (ATP) |
|  | PMPK | Phosphomethylpyrimidine kinase |
| Blon_0971 | GTPDPK | GTP diphosphokinase |
| Blon_1453 | GCDCHOLBHSe | Glycochenodeoxycholate hydrolysis by bile salt hydrolase (EC 3.5.1.24) |
|  | GCHOLBHSe | Glycocholate hydrolysis by bile salt hydrolase (EC 3.5.1.24) |
|  | TCDCHOLBHSe | Taurochenodeoxycholate hydrolysis by bile salt hydrolase (EC 3.5.1.24, EC 3.5.1.74) |
|  | TCHOLBHSe | Taurocholate hydrolysis by bile salt hydrolase (EC 3.5.1.24) |
| Blon_1585 | UP4GH | P1,P4-Bis(5-nucleosyl)-tetraphosphate nucleotidohydrolase |
| Blon_1893 | GLNSP1 | Glutamine synthetase (uaaAgld) |
|  | GLNSP2 | Glutamine synthetase (uaaGgla) |
|  | GLNSP3 | Glutamine synthetase (uaagmda) |
| Blon_1905 | BGLA | 1,4-beta-D-Glucan glucohydrolase |
|  | BGLAr | 1,4-beta-D-Glucan glucohydrolase, reversible |
|  | MADGBG | Beta-glucosidase (methyl-alpha-D-glucoside) |
|  | MBDGBG | Beta-glucosidase |
| Blon_1996 | NTPTP1 | Nucleoside triphosphate tripolyhydrolase |
| Blon_2073 | GCCb | Glycine cleavage complex |
|  | GCCc | Glycine-cleavage complex |
| Blon_2077 | THIORDXi | Hydrogen peroxide reductase (thioredoxin) |
| Blon_2246 | AMALT1 | Amylomaltase (maltotriose) |
|  | AMALT2 | Amylomaltase (maltotetraose) |
|  | AMALT3 | Amylomaltase (maltopentaose) |
|  | AMALT4 | Amylomaltase (maltohexaose) |
| Blon_2260 | FTHFCL | 5-Formethyltetrahydrofolate Cyclo-Ligase |
| Blon_2386 | TECAAE | Teichoic acid D-alanine substituted export |
|  | TECAGE | Teichoic acid glucose substituted export |
|  | TECAUE | Teichoic acid unsubstituted export |
| Blon_2390 | TDPGDH | DTDPglucose 4, 6-Dehydratase |
| Blon_2447 | FMNRx2 | FMN reductase |

**Table S3.** Summary of gene essentiality analysis

| **Gene types** | **Condition** | | | |
| --- | --- | --- | --- | --- |
|  | **Lactose** | **LNnT** | **3FL** | **6SL** |
| Essential Genes | 82 | 81 | 82 | 81 |
|  | 81 | | | |
| Non-Essential Genes | 496 | 497 | 496 | 497 |
|  | 496 | | | |
| Non-Lethal with Reduced Growth | 77 | 90 | 88 | 93 |
|  | 76 | | | |

**Table S4.** Experimental and predicted growth of *B. infantis* in different carbon sources

| **Carbon source** | **Observed growth** | **Reference** | **Result** |
| --- | --- | --- | --- |
| D-glucose | + | (1) | TP |
| Lactose | + | (2) | TP |
| Raffinose | + | (3) | TP |
| Laminarin | + | (4) | FN |
| Lacto-N-tetraose | + | (2) | TP |
| D-arabinose | - | (5) | TN |
| D-xylose | - | (5) | FP |
| Ribose | + | (5) | TP |
| Mannose | + | (5) | TP |
| Trehalose | - | (5) | FP |
| Dextrin | - | (5) | FP |
| Starch | - | (5) | TN |
| Inulin | + | (5) | TP |
| Mannitol | - | (5) | FP |
| Lacto-N-neotetraose | + | (2) | TP |
| 2-fucosyllactose | + | (2) | TP |
| 3-fucosyllactose | + | (2) | TP |
| 3-sialyllactose | + | (2) | TP |
| 6-sialyllactose | + | (2) | TP |
| Galactose | + | (1) | TP |
| Sorbitol | - | (5) | TN |
| Inositol | + | (5) | TN |
| Salicillin | + | (5) | FN |
| N-acetylglucosamine | + | (1) | TP |
| Lacto-N-biose | + | (6) | FN |
| N-acetylgalactosamine | + | (1) | TP |
| Neuraminic acid | + | (7) | TP |
| Galacto-N-biose | + | (6) | FN |
| Fucose | + | (1) | TP |
| Arginine | + | (8) | TP |
| Aspartate | + | (8) | TP |
| Asparagine | + | (8) | TP |
| Alanine | + | (8) | TP |
| Glutamate | + | (8) | TP |
| Glutamine | + | (8) | TP |
| Serine | + | (8) | TP |
| Tryptophan | + | (8) | TP |
| Threonine | + | (8) | TP |
| Serine | + | (8) | TP |
| Isoleucine | + | (8) | TP |
| Valine | + | (8) | TP |
| Leucin | + | (8) | TP |
| Cysteine | - | (8) | TN |
| Methionine | + | (8) | TP |
| Lysine | + | (8) | TP |
| Histidine | + | (8) | TP |
| Phenylalanine | + | (8) | TP |
| Tyrosine | + | (8) | TP |

**Table S5.** Characterization of essential genes based on their associated reactions

| **Subsystem** | **Genes number** | **Percentage** |
| --- | --- | --- |
| Exchange/demand reaction | 22 | 27.2% |
| Transport, extracellular | 9 | 11.1% |
| Peptide metabolism | 3 | 3.7% |
| Glycolysis/gluconeogenesis | 3 | 3.7% |
| Citric acid cycle | 3 | 3.7% |
| Glycerophospholipid metabolism | 3 | 3.7% |
| Alanine and aspartate metabolism | 3 | 3.7% |
| Pyruvate metabolism | 2 | 2.5% |
| Vitamin B2 metabolism | 2 | 2.5% |
| Arginine and proline metabolism | 2 | 2.5% |
| Folate metabolism | 2 | 2.5% |
| Pentose phosphate pathway | 2 | 2.5% |
| Cholesterol metabolism | 2 | 2.5% |
| Starch and sucrose metabolism | 2 | 2.5% |
| Nucleotide interconversion | 2 | 2.5% |
| Urea cycle | 2 | 2.5% |
| Fatty acid synthesis | 2 | 2.5% |
| Pentose and glucuronate interconversions | 2 | 2.5% |
| O-Glycan degradation | 2 | 2.5% |
| Nucleotide salvage pathway | 1 | 1.2% |
| Tetrahydrobiopterin metabolism | 1 | 1.2% |
| Fructose and mannose metabolism | 1 | 1.2% |
| Aminosugar metabolism | 1 | 1.2% |
| Thiamine metabolism | 1 | 1.2% |
| Vitamin B6 metabolism | 1 | 1.2% |
| Glycine, serine, alanine, and threonine metabolism | 1 | 1.2% |
| Respiration | 1 | 1.2% |
| Lysine metabolism | 1 | 1.2% |
| Biotin metabolism | 1 | 1.2% |
| Miscellaneous | 1 | 1.2% |

**Table S6.** Comparison of maximum specific growth rates (h^-1^) predicted by *i*LR578 under different carbon sources

| **Carbon source** | **Experimental growth rate^†^** | **Model predictions with different biomass equations^*^** | | |
| --- | --- | --- | --- | --- |
|  |  | **AGORA** | **Schöpping et al.** | **Schöpping et al.**  **(GAM adjusted)^#^** |
| Lactose | 0.171 | 0.091 | 0.208 | 0.118 |
| LNnT | 0.098 | 0.086 | 0.197 | 0.111 |
| 3FL | 0.031 | 0.069 | 0.158 | 0.088 |
| 6SL | 0.043 | 0.080 | 0.183 | 0.102 |
| Root-Mean-Square Error: __ | | 0.048 | 0.108 | 0.049 |

^†^Data from Garrido et al., (2013a) and Garrido et al., (2013b).

^*^Maximum specific growth rates were computed assuming a constant consumption rate for each substrate during the fermentation time reported in the above references. An acetate to lactate ratio of 3:2 was considered in these calculations.

^#^Biomass reaction of Schöpping et al. considering a GAM requirement of 40 mmol·gDCW^-1^ as in the AGORA biomass reaction.

**Table S7.** Comparison of maximum specific growth rates (h^-1^) predicted by *i*LR578 under different carbon sources assuming different production ratios of acetate to lactate

| **Carbon source** | **Experimental growth rate^†^** | **Ratio constraint: acetate to lactate^*^** | | |
| --- | --- | --- | --- | --- |
|  |  | **2.5 to 2** | **3 to 2** | **3.5 to 2** |
| Lactose | 0.171 | 0.089 | 0.091 | 0.093 |
| LNnT | 0.098 | 0.084 | 0.086 | 0.087 |
| 3FL | 0.031 | 0.068 | 0.069 | 0.070 |
| 6SL | 0.043 | 0.078 | 0.080 | 0.081 |
| Root-Mean-Square Error: __ | | 0.048 | 0.048 | 0.048 |

^†^Data taken from Garrido et al., (2013a) and Garrido et al., (2013b)

^*^Maximum specific growth rates were computed assuming a constant consumption rate for each substrate during the fermentation time reported in the above experiments.

**Table S8.** Comparison of biomass yields (g⋅gDW^-1^) predicted by *i*LR578 under different carbon sources integrating transcriptomcs and assuming different production ratios of acetate to lactate

| **Metabolite** | **Experimental yields** | **Ratio constraint: acetate to lactate** | | |
| --- | --- | --- | --- | --- |
|  |  | **2.5 to 2** | **3 to 2** | **3.5 to 2** |
| Lactose | 28.0 | 14.7 | 15.0 | 15.2 |
| LNnT | 21.3 | 18.3 | 18.7 | 19.0 |
| 3FL | 14.8 | 32.3 | 33.1 | 33.6 |
| 6SL | 13.1 | 23.7 | 24.3 | 24.7 |
| Root-Mean-Square Error: __ | | 12.3 | 12.6 | 12.8 |

**Table S9.** Yield of major metabolites (g⋅gDW^-1^) predicted by *i*LR578 assuming biomass maximization and a 2.5:2 production ratio of acetate to lactate under different carbon sources

| **Metabolite** | **Carbon Source** | | | |
| --- | --- | --- | --- | --- |
|  | **Lactose** | **LNnT** | **3FL** | **6SL** |
| 1,2-Propanediol | 0.00 | 0.00 | 0.79 | 0.00 |
| Acetate | 2.11 | 2.20 | 2.35 | 2.34 |
| Ethanol | 0.02 | 0.02 | 0.02 | 0.02 |
| Formate | 0.02 | 0.02 | 0.02 | 0.02 |
| Lactate | 2.57 | 2.68 | 2.87 | 2.86 |
| Succinate | 0.00 | 0.00 | 0.00 | 0.00 |

**Table S10.** Yield of major metabolites (g⋅gDW^-1^) predicted by *i*LR578 assuming biomass maximization and a 3:2 production ratio of acetate to lactate under different carbon sources

| **Metabolite** | **Carbon Source** | | | |
| --- | --- | --- | --- | --- |
|  | **Lactose** | **LNnT** | **3FL** | **6SL** |
| 1,2-Propanediol | 0.00 | 0.00 | 0.77 | 0.00 |
| Acetate | 2.27 | 2.37 | 2.53 | 2.52 |
| Ethanol | 0.02 | 0.02 | 0.02 | 0.02 |
| Formate | 0.02 | 0.02 | 0.02 | 0.02 |
| Lactate | 2.31 | 2.41 | 2.57 | 2.56 |
| Succinate | 0.00 | 0.00 | 0.00 | 0.00 |

**Table S11.** Production yields ranges of major metabolites (g⋅gDW^-1^) predicted by *i*LR578 under near-optimal growth^*^ assuming a 3.5:2 production ratio of acetate to lactate under different carbon sources

| **Metabolite** | **Carbon Source** | | | |
| --- | --- | --- | --- | --- |
|  | **Lactose** | **LNnT** | **3FL** | **6SL** |
| 1,2-Propanediol | [0 , 0] | [0 , 0] | [0.69 , 0.76] | [0 , 0] |
| Acetate | [2.29 , 2.44] | [2.35 , 2.64] | [2.51 , 2.84] | [2.51 , 2.81] |
| Ethanol | [0.02 , 0.02] | [0.02 , 0.02] | [0.02 , 0.02] | [0.02 , 0.02] |
| Formate | [0.02 , 0.02] | [0.02 , 0.02] | [0.02 , 0.02] | [0.02 , 0.02] |
| Lactate | [2 , 2.13] | [2.05 , 2.31] | [2.18 , 2.48] | [2.19 , 2.45] |
| Succinate | [0 , 0.07] | [0 , 0.08] | [0 , 0.09] | [0 , 0.08] |

^*^Near-optimal growth was defined by constraining the specific growth rate to a 99% of the computed maximum specific growth rate under each condition.

**Table S12.** Production yields ranges of major metabolites (g⋅gDW^-1^) predicted by *i*LR578 under near-optimal growth^*^ without assuming of acetate to lactate production under different carbon sources

| **Metabolite** | **Carbon Source** | | | |
| --- | --- | --- | --- | --- |
|  | **Lactose** | **LNnT** | **3FL** | **6SL** |
| 1,2-Propanediol | [0 , 0] | [0 , 0] | [0.69 , 0.76] | [0 , 0] |
| Acetate | [2.47 , 2.74] | [2.53 , 3.14] | [2.51 , 3.21] | [2.67 , 3.3] |
| Ethanol | [0.02 , 0.02] | [0.02 , 0.02] | [0.02 , 0.03] | [0.02 , 0.03] |
| Formate | [0.02 , 0.02] | [0.02 , 0.02] | [0.02 , 0.02] | [0.02 , 0.03] |
| Lactate | [1.4 , 1.91] | [1.4 , 2.04] | [1.81 , 2.53] | [1.55 , 2.21] |
| Succinate | [0 , 0.1] | [0 , 0.1] | [0 , 0.1] | [0 , 0.1] |

^*^Near-optimal growth was defined by constraining the specific growth rate to a 99% of the computed maximum specific growth rate under each condition.

**Table S13.** Comparison of various expression thresholds on the predicted biomass growth calculated with GIMME

| Threshold (%) | Predicited max. growth rate (1/h) | | | |
| --- | --- | --- | --- | --- |
|  | Lactose | LNnT | 3'FL | 6'SL |
| 20 | 0.091 | 0.084 | 0.064 | 0.077 |
| 30 | 0.085 | 0.082 | 0.063 | 0.075 |
| 40 | 0.084 | 0.081 | 0.063 | 0.075 |

**Supplementary Figures**

**
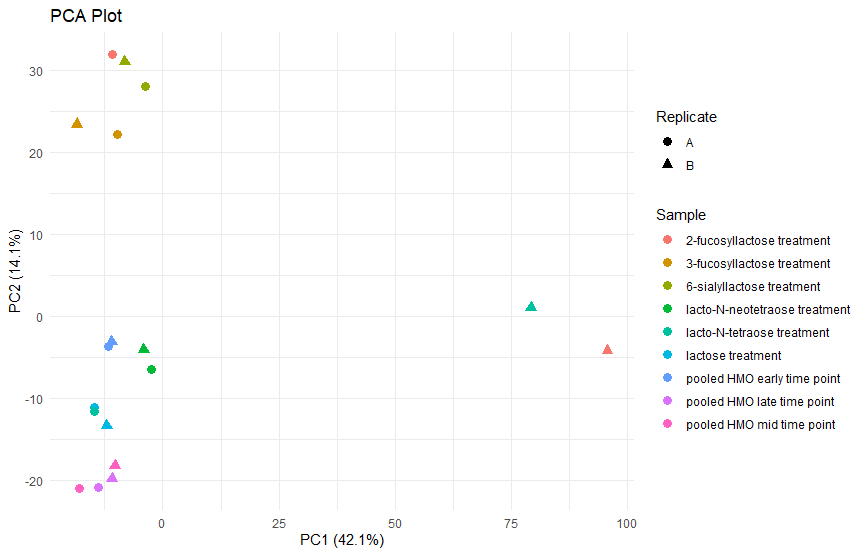
**

**Figure S1.** Principal Component Analysis (PCA) of *B. infantis* libraries without filtered *B. bifidum* reads. Treatments are color-coded whereas replicates are shown depicted by circles and triangles. The data was transformed using variance stabilization function provided by DESeq2. The two rightmost replicates behave substantially different than their counterparts. These replicates belong to the 2FL and LNT conditions, and thus, they were excluded from the analysis. The figure also includes available transcriptomics data from experiments using various HMOs sources at different growth phases, showing consistent a behavior between replicates.

**
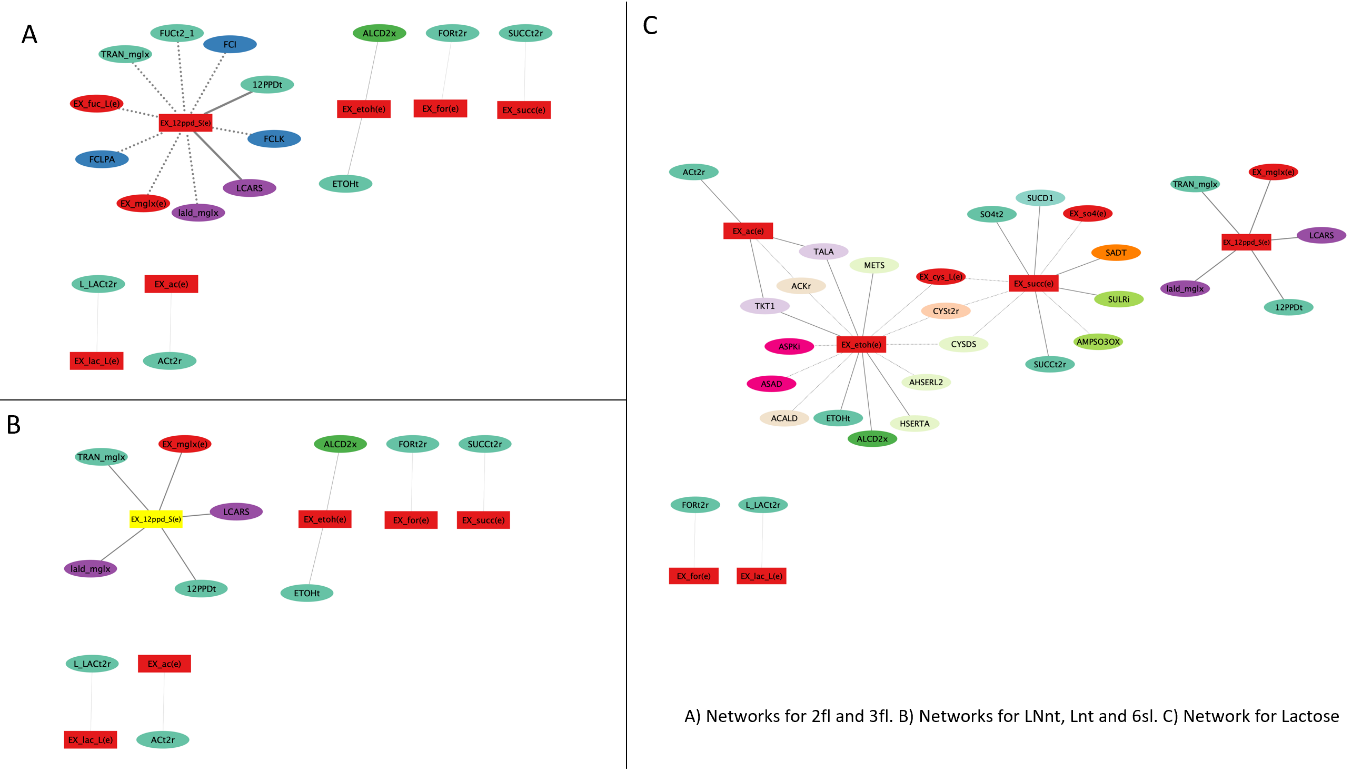
**

**Figure S2.** Coupled reaction networks for each context-specific model derived from Flux Coupling Analysis without imposing a fixed acetate to lactate production ratio. For each carbon source, major fermentation metabolites, namely: lactate, acetate, succinate, formate, ethanol, and 1,2-PD (red rectangles) are depicted linked to reactions coupled to them in each condition (colored ovals). Panels describe network generated under different carbon sources, namely: **A** 3FL, **B** 6SL and LNnT, and **C**: Lactose. The legend on the right indicates the type of reaction coupling (partial or full – dashed or solid line, respectively), edge betweenness (line thickness), and reactions subsystems (color coded). Reactions identifiers can be found in the Supplementary Data.

**References**

1. Ward RE, Niñonuevo M, Mills DA, Lebrilla CB, German JB. 2007. In vitro fermentability of human milk oligosaccharides by several strains of bifidobacteria. Mol Nutr Food Res 51:1398–1405.

2. Garrido D, Ruiz-Moyano S, Lemay DG, Sela DA, German JB, Mills DA. 2015. Comparative transcriptomics reveals key differences in the response to milk oligosaccharides of infant gut-associated bifidobacteria. Sci Rep 5:13517.

3. Perrin S, Warchol M, Grill JP, Schneider F. 2001. Fermentations of fructo-oligosaccharides and their components by Bifidobacterium infantis ATCC 15697 on batch culture in semi-synthetic medium. J Appl Microbiol 90:859–865.

4. Zhao J, Cheung PCK. 2011. Fermentation of β-Glucans Derived from Different Sources by Bifidobacteria: Evaluation of Their Bifidogenic Effect. J Agric Food Chem 59:5986–5992.

5. Sakata S, Kitahara M, Sakamoto M, Hayashi H, Fukuyama M, Benno Y. 2002. Unification of Bifidobacterium infantis and Bifidobacterium suis as Bifidobacterium longum. Int J Syst Evol Microbiol 52:1945–1951.

6. Garrido D, Kim JH, German JB, Raybould HE, Mills DA. 2011. Oligosaccharide binding proteins from bifidobacterium longum subsp. infantis reveal a preference for host glycans. PLoS One 6.

7. Idota T, Kawakami H, Nakajima I. 1994. Growth-promoting Effects of N -Acetylneuraminic Acid-containing Substances on Bifidobacteria. Biosci Biotechnol Biochem 58:1720–1722.

8. Ferrario C, Duranti S, Milani C, Mancabelli L, Lugli GA, Turroni F, Mangifesta M, Viappiani A, Ossiprandi MC, van Sinderen D, Ventura M. 2015. Exploring Amino Acid Auxotrophy in Bifidobacterium bifidum PRL2010. Front Microbiol 6:1331.
