# Supplementary material for: Genome-scale metabolic modeling of the human milk oligosaccharide utilization by Bifidobacterium longum subsp. infantis
Source: mSystems. 2024 Feb 16;9(3):e00715-23. doi: 10.1128/msystems.00715-23 (PMC10949479; doi:10.1128/msystems.00715-23)
Supplement: Legend — for Supplemental Data. [file msystems.00715-23-s0003.docx]

**LEGEND TO SUPPLEMENTARY DATA**

**Genes:** List of genes (locus tags) in final model iLR578

**Metabolites:** List of metabolites part of metabolic model iLR578

**Reactions:** List of reactions in iLR578

**Modified rxns from AGORA 1.03:** List of eliminated or added reactions with respect to original AGORA model v1.03

**Medium definition:** Metabolites present in simulated modified Lactobacilli MRS

**Transcriptomics (norm counts):** Gene expression values (normalized counts) for dataset used in this study.

**GIMME FBA:** Flux Balance Analysis results run with Gene Inactivity Moderated by Metabolism and Expression GIMME.

**Ubiquitously Expressed Genes:** Prediction of Reactions that should be expressed based on transcriptomics data according to essentiality analysis.

**Single-gene KO:** Essentiality analysis of each gene (locus_tag) in iLR578, assessed as knock-out impact in growth rate

**Essentiality analysis:** List of genes in iLR578 predicted to be essential and common to all carbon sources
